# Characterising Eye Movement Events with an Unsupervised Hidden Markov Model

**DOI:** 10.16910/jemr.15.1.4

**Published:** 2022-06-28

**Authors:** Malte Lüken, Šimon Kucharský, Ingmar Visser

**Affiliations:** Department of Psychology, University of Amsterdam, The Netherlands; *These authors contributed equally

**Keywords:** eye tracking, eye movements, event classification, event detection, dependent mixture models, fixations, saccades, post-saccadic oscillations, smooth pursuits

## Abstract

Eye-tracking allows researchers to infer cognitive processes from eye movements that are classified into distinct events. Parsing the events is typically done by algorithms. Here we aim at developing an unsupervised, generative model that can be fitted to eye-movement data using maximum likelihood estimation. This approach allows hypothesis testing about fitted models, next to being a method for classification. We developed gazeHMM, an algorithm that uses a hidden Markov model as a generative model, has few critical parameters to be set by users, and does not require human coded data as input. The algorithm classifies gaze data into fixations, saccades, and optionally postsaccadic oscillations and smooth pursuits. We evaluated gazeHMM’s performance in a simulation study, showing that it successfully recovered hidden Markov model parameters and hidden states. Parameters were less well recovered when we included a smooth pursuit state and/or added even small noise to simulated data. We applied generative models with different numbers of events to benchmark data. Comparing them indicated that hidden Markov models with more events than expected had most likely generated the data. We also applied the full algorithm to benchmark data and assessed its similarity to human coding and other algorithms. For static stimuli, gazeHMM showed high similarity and outperformed other algorithms in this regard. For dynamic stimuli, gazeHMM tended to rapidly switch between fixations and smooth pursuits but still displayed higher similarity than most other algorithms. Concluding that gazeHMM can be used in practice, we recommend parsing smooth pursuits only for exploratory purposes. Future hidden Markov model algorithms could use covariates to better capture eye movement processes and explicitly model event durations to classify smooth pursuits more accurately.

## Introduction

Eye-tracking is often used to study cognitive processes involving attention and information search based on recorded gaze position (43). Before these processes can be studied, the raw gaze data is classified into events that are distinct in their physiological patterns (e.g., duration), underlying neurological mechanisms, or cognitive functions (30). Basic events are fixations, saccades, smooth pursuits, and post-saccadic oscillations (PSOs). Classifying raw eye-tracking data into these events reduces their complexity and is usually the first step towards cognitive interpretation (41). The classification is typically done by algorithms, which is considered faster, more objective, and reproducible compared to human coding (1). Hein and Zangemeister (17) give a comprehensive overview of different classification algorithms (for a structured review on classifying saccades, see also Stuart et al., 49).

The aim of the current study is to develop a generative, unsupervised model for characterising, describing, and understanding eye movement data. Below we discuss the requirements for such a model. One such requirement is obviously that it can reliably classify eye movement events.

To motivate our decision to add another algorithm to this array of classification tools, it is useful to briefly discuss the properties and goals of those tools. On one hand, many classification algorithms use non-parametric methods to differentiate between eye movement events^.^ We use the terms classification and event classification throughout this paper but see discussion about the appropriateness of those terms as compared with event detection in (19).

A classic example is the “Velocity-threshold” algorithm (47), which classifies samples with a velocity above a fixed threshold as saccades (see also 28; 29; 36). On the other hand, many parametric methods have been developed recently. Some of them require human-labeled training data as input and can therefore be termed as supervised (15). For example, Bellet et al. (4) trained a convolutional neural network (CNN) on eye-tracking data from humans and macaques and achieved saccade classifications that were highly similar to those of human coders (for other supervised algorithms, see 48; 58; 59). Due to their high agreement with human coders, one might call the supervised approaches “state-of-the-art”. However, the requirement of labeled training data is a disadvantage of supervised methods because the labeling process can easily become costly and time-consuming (58). More importantly, supervised methods also (implicitly) treat human-labeled training data as a reliable gold standard, an assumption that may be unwarranted (see discussion in 21). The reliance on training data also makes supervised methods inflexible: When test data strongly deviates from the training data, the classification performance can decrease substantially (e.g., 48). Furthermore, when the required events for test data differ from the hand-coded events in the training data, the latter would need to be recoded, causing additional costs.

In contrast, unsupervised classification algorithms do not require labeled training input. Instead, they learn parameters from the characteristics of the data themselves (15). In consequence, they are also more flexible in classifying data from different individuals, tasks, or eye-trackers (e.g., 18; 22).

Besides discriminating between supervised and unsupervised methods, algorithms can vary in whether they are explicitly modeling the data generating process and are thus able to simulate new data. To our knowledge, these generative models have been rarely used to classify eye movement data (cf. 35; 55). Classifiers with generative assumptions have the advantage that their parameters can be easily interpreted in terms of the underlying theory. In the context of eye movements, they can also help to explain or confirm observed phenomena: For instance, their parameters can indicate that oscillations only occur after but not before saccades. When the goal is to understand eye movement events and improve their classification based on this understanding, this aspect is an advantage over non-parametric or supervised methods. Moreover, generative models can challenge common theoretical assumptions and bring up new research questions (12). For example, they might suggest that oscillations also occur before saccadic eye movements (as mentioned in Nyström & Holmqvist, 36) or that the assumption that eye movements are discrete events (e.g., saccades and PSOs cannot overlap) does not hold (as discussed in Andersson et al., 1).

We argue that the recent focus on supervised approaches misses an important facet of eye movement event classification: Supervised methods are trained on human-labeled data and can predict human classification well. This is an important milestone for applicants that are interested in automating human classification. However, since human classification may not be as reliable, valid, and objective as assumed (1; 21), supervised approaches will also reproduce these flaws. Instead, we suggest taking a different avenue and developed an unsupervised, generative algorithm to set a starting point for more explicit parametric modeling of common eye movement events (cf. 35). By relying on likelihood-based goodness-of-fit measures, we aim to achieve a classification that reaches validity through model comparison instead of making the classification more human-like. A model-based approach can also improve the reliability because it will lead to the same classification given the correct settings, whereas human annotation can depend on implicit, idiosyncratic thresholds that may be hard to reproduce (see 21).

One class of generative models that are used in eye movement classification are HMMs. They estimate a sequence of hidden states (i.e., a discrete variable that cannot be directly observed) that evolves parallel to the gaze signal. Each gaze sample depends on its corresponding state. Each state depends on the previous but not on earlier states of the sequence (60). Further, HMMs can be viewed as unsupervised models that can learn the hidden states and parameters of the emission process from the observed data alone, and as such do not in principle need labeled training data. They are suitable models for eye movement classification because the hidden states can be interpreted as eye movement events and gaze data are dependent time series (i.e., one gaze sample depends on the previous). HMMs can be applied to individual or aggregated data (or both, see 22) and are thus able to adapt well to interindividual differences in eye movements.

On this basis, several classification algorithms using HMMs have been developed: One instance is described in Salvucci and Goldberg (41) and combines the HMM with a fixed threshold approach (named “Identification by HMM” [I-HMM]). Samples are first labeled as fixations or saccades, depending on whether their velocity exceeds a threshold, and then reclassified by the HMM. Pekkanen and Lappi (38) developed an algorithm that filters the position of gaze samples through naive segmented linear regression (NSLR). The algorithm uses an HMM to parse the resulting segments into fixations, saccades, smooth pursuits, and PSOs based on their velocity and change in angle (named NSLR-HMM). Another version by Mihali et al. (35) uses a Bayesian HMM to separate microsaccades (short saccades during fixations) from motor noise based on sample velocity (named “Bayesian Microsaccade Detection” [BMD]). Moreover, Houpt et al. (22) applied a hierarchical approach developed by Fox and colleagues that describes sample velocity and acceleration through an autoregression (AR) model, computes the regression weights through an HMM, and estimates the number of events with a beta-process (BP) from the data (named BP-AR-HMM).

Several studies have tested the performance of HMM algorithms against other

classification methods: I-HMM has been deemed as robust against noise, behaviorally accurate, and showing a high sample-to-sample agreement to human coders (1; 24; 41). However, the agreement was lower when compared to an algorithm using a Bayesian mixture model (23; 50). NSLR-HMM showed even higher agreement to human coding than I-HMM (38) but was outperformed for saccades by the CNN algorithm by Bellet et al. (4).

In sum, HMMs seem to be a promising method for classifying eye movements in unsupervised settings. Nevertheless, the existing HMM algorithms each have at least one aspect in which they could be improved.

First, I-HMM relies on setting an appropriate threshold to determine the initial classification, which can distort the results (8; 24; 45). Second, the current implementation of NSLR-HMM requires human-coded data, which narrows its applicability to applications where supervised methods are also an option. It also inheres fixed parameters that prevent the algorithm to adapt to individual or task-specific signals. Third, BMD limits the classification to microsaccades which are irrelevant in many applications and sometimes even considered as noise (10). The opposite problem was observed for BP-AR-HMM: It tends to estimate an unreasonable number of events from the data of which many are considered as noise events (e.g., blinks). Therefore, the authors suggest using it as an exploratory tool followed by further event classification (22).

### Goals

The goal of the project reported in this article is to move towards generative models of eye movement events. The purpose of generative models is to bring better understanding of the events they describe in a fully statistical framework, which enables likelihood-based comparisons and hypothesis tests, or to generate novel hypotheses. Such models can be also used for classification, even though that may not be their only or primary application.

In this article, we present a novel model of eye movement events, named gazeHMM, that relies on an HMM as a generative model.

The first step in developing a generative model that can be also used as a statistical model (e.g., to be fit to data), is to ensure its computational consistency, that is, whether the model is able to recover parameter values that were used to generate the data. Second, as classification is one of the possible applications of such model, it is important to evaluate the classification performance and ensure that the model does reasonably well identifying the eye movement events it putatively describes. We believe these two questions are the minimal requirements of a generative model in the current setting, and the current article brings just that — evaluation of the basic characteristics of a generative model that we developed.

Table 1 presents a selection of recently developed classification algorithms (i.e., the “state-of-the-art”) and highlights the contribution of gazeHMM for the purpose of eye movement classification: First, our algorithm uses an unsupervised classifier and thus does not require human-coded training data. This independence also allows gazeHMM to adapt well to interindividual differences in gaze behavior. Second, gazeHMM uses a parametric model (i.e., an HMM) and relies on maximum likelihood estimation, which enables model comparisons and testing parameter constraints. This property has been rarely used in eye movement event models. Third, it classifies the most relevant eye movement events, namely, fixations, saccades, PSOs, and smooth pursuits. Additionally, gazeHMM gives the user the option to only classify the first two or the first three of these events, a feature that most other algorithms do not have. As a minor goal, we aimed to reduce the number of thresholds which users must set to a minimum.

The following section describes gazeHMM and the underlying generative model in detail. Then, we present the parameter recovery of the HMM and show how the algorithm performs compared to other eye movement event classification algorithms concerning the agreement to human coding. Importantly, we did not compare gazeHMM to supervised algorithms due to the training requirements of these methods. Finally, we discuss these results and propose directions in which gazeHMM and other HMM algorithms could be improved.

## Developing gazeHMM

**Figure 1.**
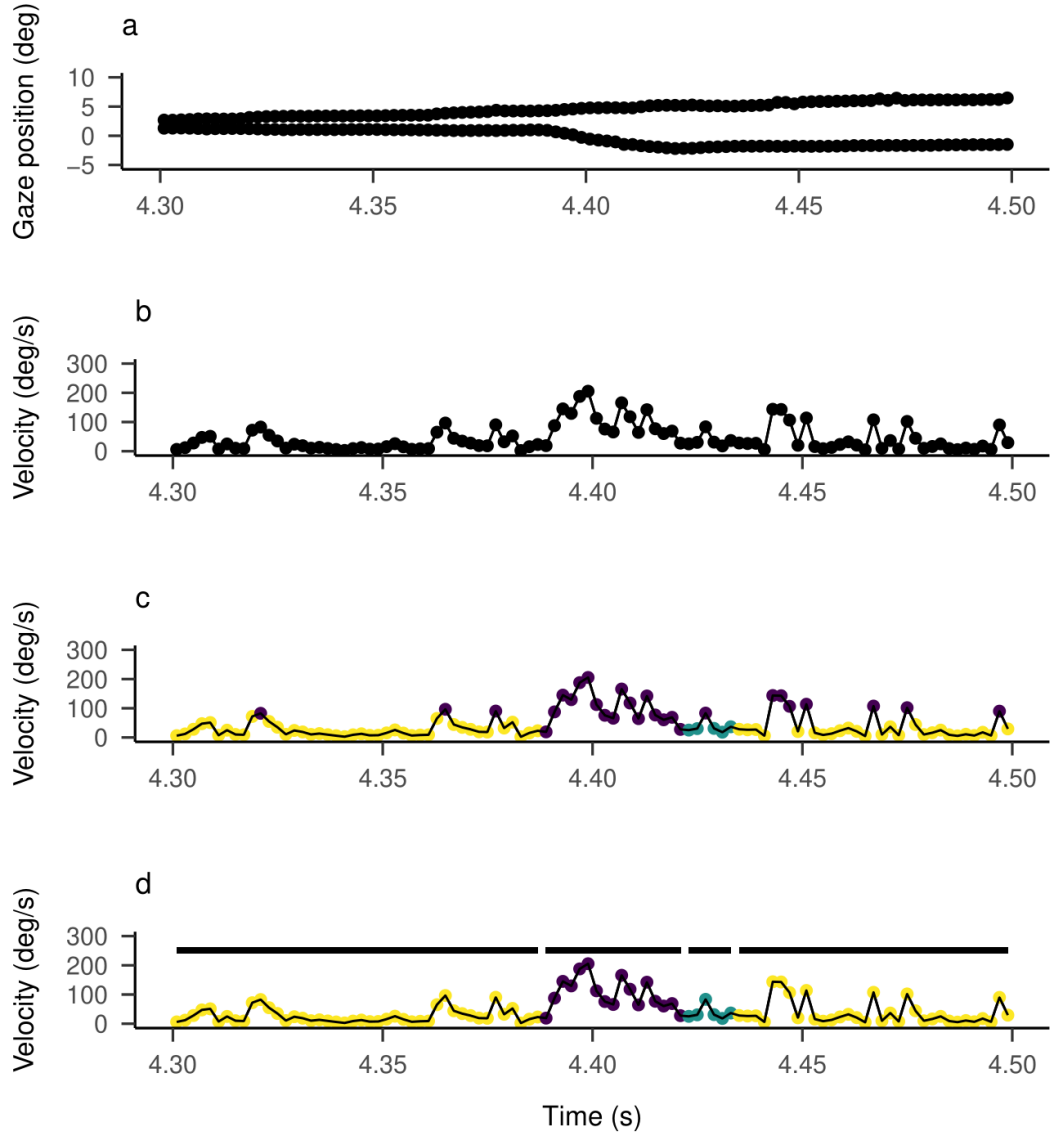
Example Workflow for Eye Movement Event Classification Algorithms.

Note. Workflow description: (a) the raw gaze signal in x (upper line) and y (lower line) coordinates; (b) the raw gaze signal is filtered and transformed into a velocity signal; (c) samples are classified as events (indicated by colors), and (d) relabeled. Sequences of samples belonging to the same event are merged (indicated by black segments). Data from Andersson et al. (1).

As illustrated in Figure 1, most eye movement event classification algorithms consist of three steps (cf. 18): During preprocessing, features (such as velocity and acceleration) are extracted from the raw gaze positions. Often, a filtering or smoothing procedure is applied to the data, before or after the transformation, to separate the gaze signal from noise and artifacts (46). Then follows the classification, depending on the method and settings of the algorithm, each sample is labeled as a candidate for one of the predefined events. Lastly, as part of the postprocessing, the algorithm decides which candidates to accept, relabel, or merge (18; 24). Note that Hessels et al. (18) called step two the search rule and step three the classification rule. For non-parametric methods, this distinction might be accurate. However, for parametric methods, calling step two "classification" is more appropriate since the probabilistic classification is done here. Step three usually consists of some heuristic relabeling and correcting for classification errors.

### Preprocessing

Algorithms require variables that describe gaze data (hereafter called eye movement features) to classify them into events. Many eye movement features have been proposed and used in previous algorithms (for examples, see 1; 59), but most of them rely on thresholds or window ranges that have to be set by the user (e.g., the distance between the mean position in a 100 ms window before and after each sample, see 37). This can be problematic because such parameters are often **Table 1** set without theoretical justification and differ substantially between features or heavily depend on the eye-tracker’s characteristics (e.g., sampling frequency, 1). In gazeHMM, we used velocity, acceleration, and sample-to-sample angle (synonymous to relative or change in angle Larsson et al., 28) because they belong to the most basic features which do not require additional parameter settings.

**Table 1.**
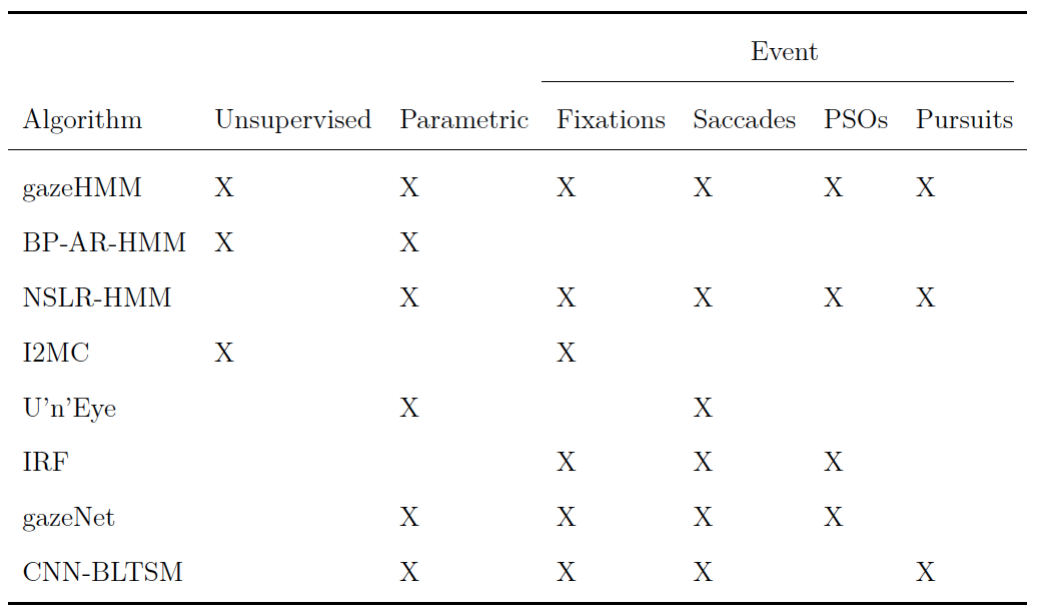
Recently Developed Algorithms for Eye Movement Classification.

Note. X means that an algorithm has the respective property or classifies the respective event. BP-AR-HMM = beta-process autoregressive HMM (22); NSLR-HMM = naive segmented linear regression HMM (38); I2MC = identification by two-means clustering (18); U’n’Eye by Bellet et al. (4); IRF = identification by random forest (59); gazeNet by Zemblys et al. (58); CNN-BLTSM = convolutional neural network bidirectonal long short-term memory (48).

Theoretically, these three features should separate eye movement events, depending on one’s definitions (19). In the present work, we assume eye-tracking applications with fixed head position (chin-rest), gazing at a fixed display with a stationary eye-tracker. Fixations typically show samples with low velocity and acceleration. Due to tremor, we assume that the angle between samples should not follow any direction but a uniformly random walk. In contrast, saccade samples usually have a high velocity and acceleration and roughly follow the same direction. PSO samples tend to have moderate velocity and high acceleration since they occur between saccades and low-velocity events (28; 29). They can be specifically distinguished by their change in direction clustered around 180 degrees (38). Importantly, the feature distribution during oscillations depends on the resolution of the gaze recording: Eye-trackers with higher sampling frequency yield more changes in direction and more samples in between those changes. Those samples in between typically follow the same direction. Thus, with high sampling frequencies, PSO samples might also cluster around a sample-to-sample angle of zero with outliers around 180 degrees. Lastly, smooth pursuit samples have a moderate velocity but low acceleration (due to the smoothness) and like saccades, they follow a similar direction (28; 30). Other algorithms focus exclusively on classifying microsaccades (e.g., 35), but as stated earlier, these events were not in the scope of gazeHMM. The velocity and acceleration signals are computed from the raw gaze position by using a Savitzky-Golay filter (similar to 36; 42). The sample-to-sample angle is calculated as:

**(1):**
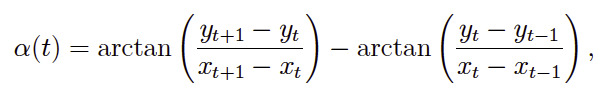


with α(t) := α(t) + 2π for α(t) < 0, and is therefore bound between 0 and 2π. Most of the missing data in eye movement data are due to blinks. In gazeHMM, we do not consider blinks as an additional event but rather as another source of noise. Therefore, the user can provide an indicator for samples that should be labeled as blinks (e.g., based on automated blink detection through the eye-tracker). Often, eye-trackers record a few samples with unreasonably high velocity and acceleration before losing the pupil signal when a blink occurs. Since these samples could distort the classification of saccades in the HMM, gazeHMM removes them heuristically. Before classifying the samples, it sets all samples within 50 ms before and after blink samples as missing. We note that this arbitrary setting is undermining our development goal of requiring as few user settings as possible. However, when we included blinks in the generative model itself, the classification of the other events became worse. Thus, we justify the heuristic blink removal by its accuracy, simplicity, and practicality. Furthermore, we experienced during the development that the default setting of 50 ms was appropriate for all data we examined.

## The Generative Model

We denote the three eye movement features by X, Y , and Z. Each feature was generated by a hidden state variable S. Given S, the HMM treats X, Y , and Z as conditionally independent. Conditional independence might not accurately resemble the relationship between velocity and acceleration (which are naturally correlated). This step was merely taken to keep the HMM simple and identifiable. In gazeHMM, S can take one of two, three, or four hidden states. By selecting appropriate default starting values for the states (see Table 4), the algorithm is nudged to associate them with the same eye movement events. We remark that gazeHMM does not guarantee a consistent correspondence between states and events (see the phenomenon of label switching in the simulation study discussion). However, when applying gazeHMM to eye movement data, we did not encounter any problems in this regard. Moreover, gazeHMM comes with tools for a ‘sanity check’ to confirm whether expected and estimated state characteristics match (i.e., the HMM converged to an appropriate solution). Given correct identification, the first state represents fixations, the second saccades, the third PSOs, and the fourth smooth pursuits. Thus, users can choose whether they would like to classify only fixations and saccades, or additionally PSOs and/or smooth pursuits. HMMs can be described by three submodels: An initial state model, a transition model, and a response model. The initial state model contains probabilities for the first state of the hidden sequence ρi = P(S1 = i), with i denoting the hidden state. In gazeHMM, the initial states are modeled by a multinomial distribution. The evolution of the sequence is in turn described by the transition model, which comprises the probabilities for transitioning between different states in the HMM. Typically, probabilities to transition from state i to j, aij = P(St+1 = j|St = i), are expressed in matrix form (52):

**(2):**
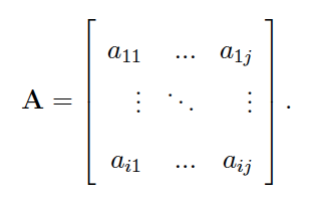


Again, the transition probabilities for each state are modeled by multinomial distributions. The response model encompasses distributions describing the response variables for every state in the model. Previous algorithms have used Gaussian distributions to describe velocity and acceleration signals (sometimes after log-transforming them). However, several reasons speak against choosing the Gaussian: First, both signals are usually positive (depending on the computation). Second, the distributions of both signals appear to be positively skewed conditionally on the states, and third, to have variances increasing with their mean. Thus, instead of using the Gaussian, it could be more appropriate to describe velocity and acceleration with a distribution that has these three properties. In gazeHMM, we use gamma distributions with a shape and scale parametrization for this purpose:

**(3):**
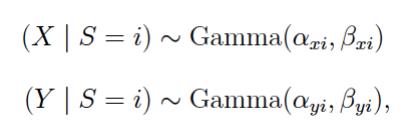


with i denoting the hidden state. When we developed gazeHMM, the gamma distribution appeared to fit eye movement data well, but we also note that it might not necessarily be the best fitting distribution for every type of eye movement data. We assume that the best fitting distribution will depend on the task, eye-tracker, and individual (see discussion). We emphasize that gazeHMM does not critically depend on the choice of distribution and other distributions than the gamma can be readily included in the model, for example the log-normal has the same required properties of being positive and positively skewed. To model the sample-to-sample angle, we pursued a novel approach in gazeHMM: A mixture of von Mises distributions (with a mean and concentration parameter) and a uniform distribution:

**(4):**
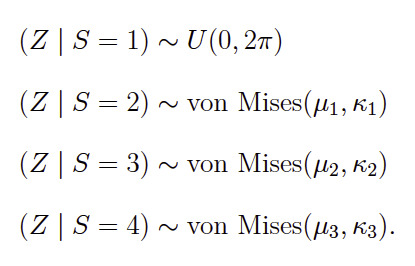


Both the distributions and the feature operate on the full unit circle (i.e., between 0 and 2π), which should lead to symmetric distributions. The von Mises is a maximum entropy distribution on a circle under a specified location and concentration, and can be considered an analogue to the Gaussian distribution in circular statistics (34). Because we assume fixations to change their direction similar to a uniformly random walk (28; 29), their sample-to-sample angle can be modeled by a uniform distribution. Thus, the uniform distribution should distinguish fixations from the other events. Taking all three submodels together, the joint likelihood of the observed data and hidden states can be expressed as:

**(5):**
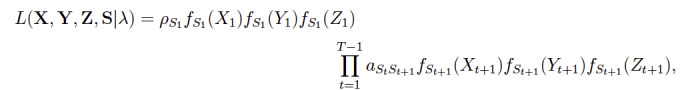


with λ denoting the vector containing the initial state and transition probabilities as well as the response parameters. By summing over all possible state sequences, the likelihood of the data given the HMM parameters becomes (52):

**(6):**
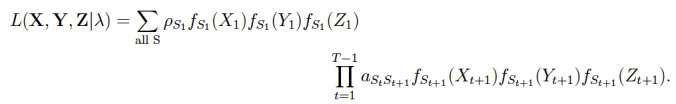


The parameters of the HMM are estimated through maximum likelihood using an expectation-maximization (EM) algorithm (9; McLachlan & Krishnan, 1997). The EM algorithm is generally suitable to estimate likelihoods with missing variables. For HMMs, it imputes missing with expected values and iteratively maximizes the joint likelihood of parameters conditional on the observed data and the expected hidden states (i.e., eye movement events Visser, 52). When evaluating the likelihood of missing data, gazeHMM integrates over all possible values, which results in a probability density of one. The sequence of hidden states is estimated through the Viterbi algorithm (13; 54) by maximizing the posterior state probability. Parameters of the response distributions (except for the uniform distribution) are optimized on the log-scale (except for the mean parameter of the von Mises distribution) using a spectral projected gradient method (6) and Barzilai-Borwein step lengths (3). The implementation in depmixS4 allows to include time-varying covariates for each parameter in the HMM. In gazeHMM, no such covariates were included and thus, only intercepts were estimated for each parameter.

### Postprocessing

After classifying gaze samples into states, gazeHMM applies a postprocessing routine to the estimated state sequence. We implemented this routine because in a few cases, gazeHMM would classify samples that were not following saccades as PSOs.

Constraining the probabilities for nonsaccade events to turn into PSOs to zero often caused PSOs not to appear in the state sequence at all. Moreover, gazeHMM does not explicitly control the duration of events in the HMM which occasionally led to unreasonably short events. Thus, the postprocessing routine heuristically compensates for such violations. This routine relabels one-sample fixations and smooth pursuits, saccades with a duration below a minimum threshold (here: 10 ms), and PSOs that follow nonsaccade events.

Samples are relabeled as the state of the previous event. Finally, samples initially indicated as missing are labeled as noise (including blinks) and event descriptives are computed (e.g., fixation duration). The algorithm is implemented in R (version: 3.6.3 40) and uses the packages signal (32) to compute velocity and acceleration signals, depmixS4 (53) for the HMM, CircStats (33) for the von Mises distribution, and BB (51) for Barzilai-Borwein spectral projected gradient optimization. The algorithm is available on GitHub (https://github.com/maltelueken/gazeHMM). We conducted a parameter recovery study that is also available on GitHub (https://github.com/maltelueken/gazeHMM_validation) showing that the model recovers parameters well when the noise level is not too high.

## Simulation Study

**Figure 2.**
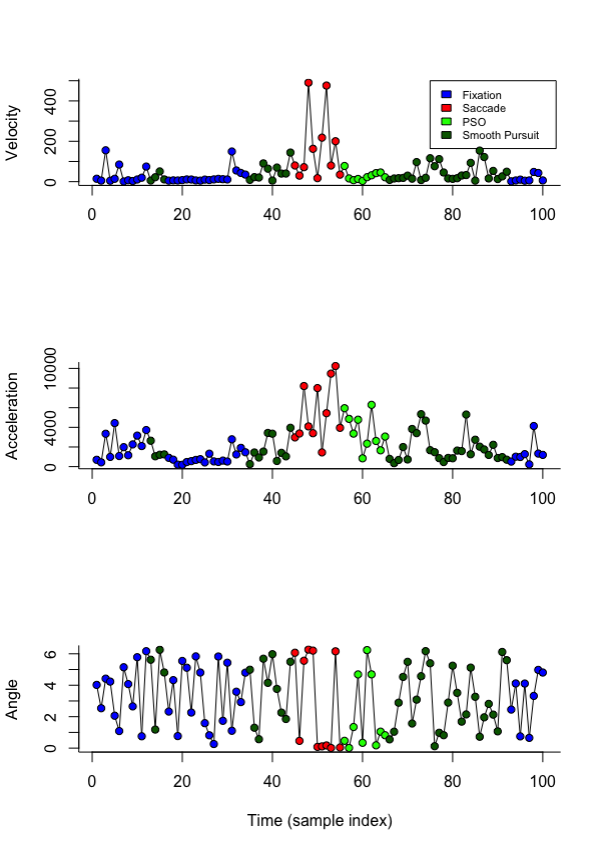
Example of Data Simulated from gazeHMM.

As a first step to validate the model, we need to ensure that fitting the model to the data results in recovering the properties of the underlying data generating process. The standard procedure in computational modeling is conducting parameter recovery study (16). Although this step is crucial when developing new models, it is often not done or goes unreported in eye-tracking literature. To counter this trend, we report a simulation study we conducted to assess the recovery of parameter values and state sequences. The design and analysis of the study were preregistered on the Open Science Framework (https://doi.org/10.17605/OSF.IO/VDJGP). The majority of this section is copied from the preregistration (with adapted tenses). The study was divided in four parts. Here, we only report the first two parts, which investigate the influence of parameter variation and adding noise to generated data on recovery. The other two parts, which address starting values and missing data, can be found in the supplementary material https://github.com/maltelueken/gazeHMM_validation). The HMM repeatedly generated data with a set of parameters (henceforth: true parameter values). An example of the simulated data is shown in Figure 2. The same model was applied to estimate the parameters from the generated data (henceforth: estimated parameter values). We compared the true with the estimated parameter values to assess whether a parameter was recovered by the model. Additionally, we contrasted the true states of the HMM with the estimated states to judge how accurately the model recovered the states that generated the data.

### Starting Values

The HMM always started with a uniform distribution for the initial state and state transition probabilities. Random starting values for the estimation of shape, scale, and concentration parameters were generated by gamma distributions with a shape parameter of α_start_ = 3 and β_start;i_ = θ_i_/2, with θ_i_ being the true value of the parameter to be estimated in simulation i ∈ (1,...,I). This setup ensured that the starting values were positive, their distributions were moderately skewed, and the modes of their distributions equaled the true parameter values. The mean parameters of the von Mises distribution always started at their true values.

### Design

In the first part, we varied the parameters of the HMM. For models with k ∈ {2,3,4} states, q ∈ {10,15,20} parameters were manipulated, respectively. For each parameter, the HMM generated 100 data sets with N = 2500 samples, and the parameter varied in a specified interval in equidistant steps. This resulted in 100 × (10 + 15 + 20) = 4500 recoveries. Only one parameter alternated at once, the other parameters were set to their default values. All parameters of the HMM were estimated freely (i.e., there were no fixed parameters in the model). We did not manipulate the initial state probabilities because these are usually irrelevant in the context of eye movement classification. For the transition probabilities, we only simultaneously changed the probabilities for staying in the same state (diagonals of the transition matrix) to reduce the complexity of the simulation. The leftover probability mass was split evenly between the probabilities for switching to a different state (per row of the transition matrix). Moreover, we did not modify the mean parameters of the von Mises distributions: As location parameters, they do not alter the shape of the distribution and they are necessary features for the HMM to distinguish between different states. We defined approximate ranges for each response variable (see supplementary material) and chose true parameter intervals and default values so that they produced samples that roughly corresponded to these ranges. Tables 2 and 3 show the intervals and default values for each parameter in the simulation. Parameters were scaled down by factor 10 (compared to the reported ranges) to improve fitting of the gamma distributions. We set the intervals for shape parameters of the gamma distributions for all events to [1,5] to examine how skewness influenced the recovery (shape values above five approach a symmetric distribution). The scale parameters were set so that the respective distribution approximately matched the assumed ranges. Since the concentration parameters of the von Mises distribution are the inverse of standard deviations, they were varied on the inverse scale. In the second part, we manipulated the sample size of the generated data and the amount of noise added to it.

**Table 2.**
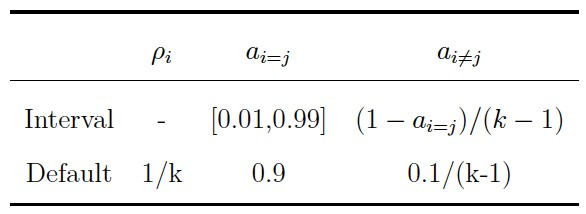
Intervals and Default Parameter Values for the Transition Model in the Simulation Study.

Note. The initial state probability is denoted by ρ_i_. The transition probability for staying in the same state is denoted by a_i=j_ and the probability for switching to a different state by a_i̸=j_. The number of states in the model is denoted by k.

The model parameters were set to their default values. For models with k ∈ {2,3,4} states and sample sizes of N ∈ {500,2500,10000}, we generated 100 data sets (100 × 3 × 3 = 900 recoveries). These sample sizes roughly match small, medium, and large eye-tracking data sets for a single participant and trial (e.g., with a frequency of 500 Hz, the sample sizes would correspond to recorded data with lengths of 1 s, 5 s, and 20 s, respectively). To simulate noise, we replaced velocity and acceleration values y with draws from a gamma distribution with α_noise_ = 3 and β_noise_ = (y/2)τ_noise_ with τ_noise_ ∈ [1,5] varying between data sets. This procedure ensured that velocity and acceleration values remained positive and were taken from moderately skewed distributions with modes equal to the original values. To angle, we added white noise from a von Mises distribution with µ_noise_ = 0 and κ_noise_ ∈ 1/[0.1,10] varying between data sets. τ_noise_ and κ_noise_ were increased simultaneously in equidistant steps in their intervals.

**Table 3.**
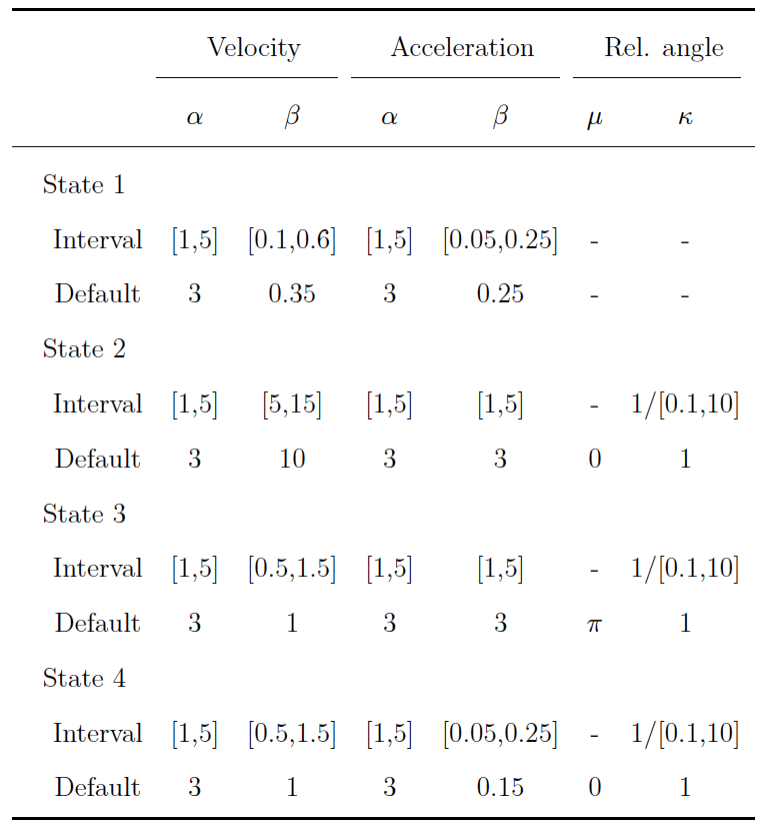
Intervals and Default Parameter Values for the Response Model in the Simulation Study.

Note. Shape parameters are denoted by α, scale parameters by β, mean parameters by μ, and concentration parameters by κ. The default values for the uniform distribution in state one were min = 0 and max = 2π.

### Data Analysis

For each parameter separately, we calculated the root median square proportion deviation (RMdSPD; analogous to root median square percentage errors, see Hyndman & Koehler, 2006) between the true and estimated parameter values:

**(7):**
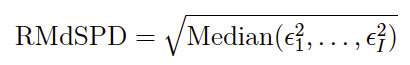


**(8):**
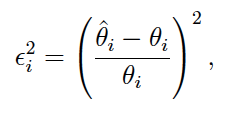


where θ_i_ is the true parameter value and θ^ˆ^_i_ is the estimated parameter value for simulation i ∈ (1,...,I), respectively. Even though it was not explicitly mentioned in the preregistration, this measure is only appropriate when θ_i_ /= 0. This was not the case for some mean parameters of the von Mises distributions. In those cases, we used θ_i_ = 2π instead. We treated RMdSPD < 0.1 as good, 0.1 ≤ RMdSPD < 0.5 as moderate, and RMdSPD ≥ 0.5 as bad recovery of a parameter. By taking the median, we reduced the influence of potential outliers in the estimation and using proportions enabled us to compare RMdSPD values across parameters and data sets.

Additionally, we applied a bivariate linear regression with the estimated parameter values as the dependent and the true parameter values as the independent variable to each parameter that has been varied on an interval in part one. Regression slopes closer to one indicated that the model better captured parameter change. Regression intercepts different from zero reflected a bias in parameter estimation.

To assess state recovery, we computed Cohen’s kappa (for all events taken together, not for each event separately) as a measure of agreement between true and estimated states for each generated data set. Cohen’s kappa estimates the agreement between two classifiers accounting for the agreement due to chance. Higher kappa values were interpreted as better model accuracy. We adopted the ranges proposed by Landis and Koch (26) to interpret kappa values. Models that could not be fitted were excluded from the recovery.

### Results

#### Parameter Variation

In the first part of the simulation, we examined how varying the parameters in the HMM affected the deviation of estimated parameters and the accuracy of estimated state sequences. For the two-state HMM, the recovery of parameters and states was nearly perfect (all RMdSPDs < 0.1, intercepts and slopes of regression lines almost zero and one, respectively, and Cohen’s kappa close to 1). Therefore, we chose to include the respective figures in the supplementary material.

**Figure 3.**
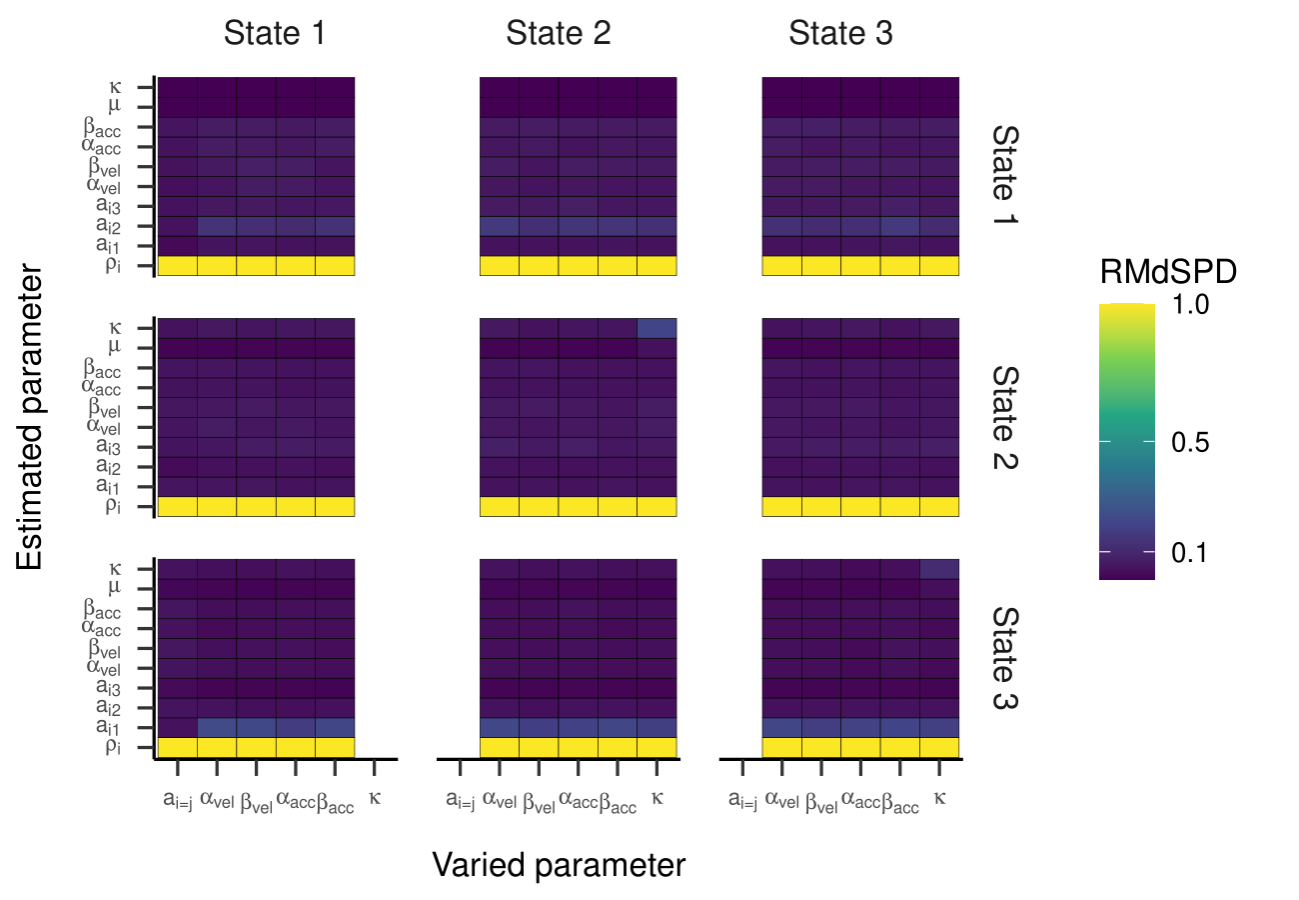
RMdSPD Between True and Estimated Parameters of the Three-State HMM in Part One of the Simulation.

For the HMM with three states, the RMdSPD is shown in Figure 3. When response parameters (other than a_i=j_) were manipulated, the RMdSPDs for a_12_ and a_31_ were consistently between 0.1 and 0.5. Varying κ in states two and three led to RMdSPDs between 0.1 and 0.5 in the respective states, which we interpreted as moderate recovery. Otherwise, RMdSPDs were consistently lower than 0.1, indicating good recovery.

**Figure 4.**
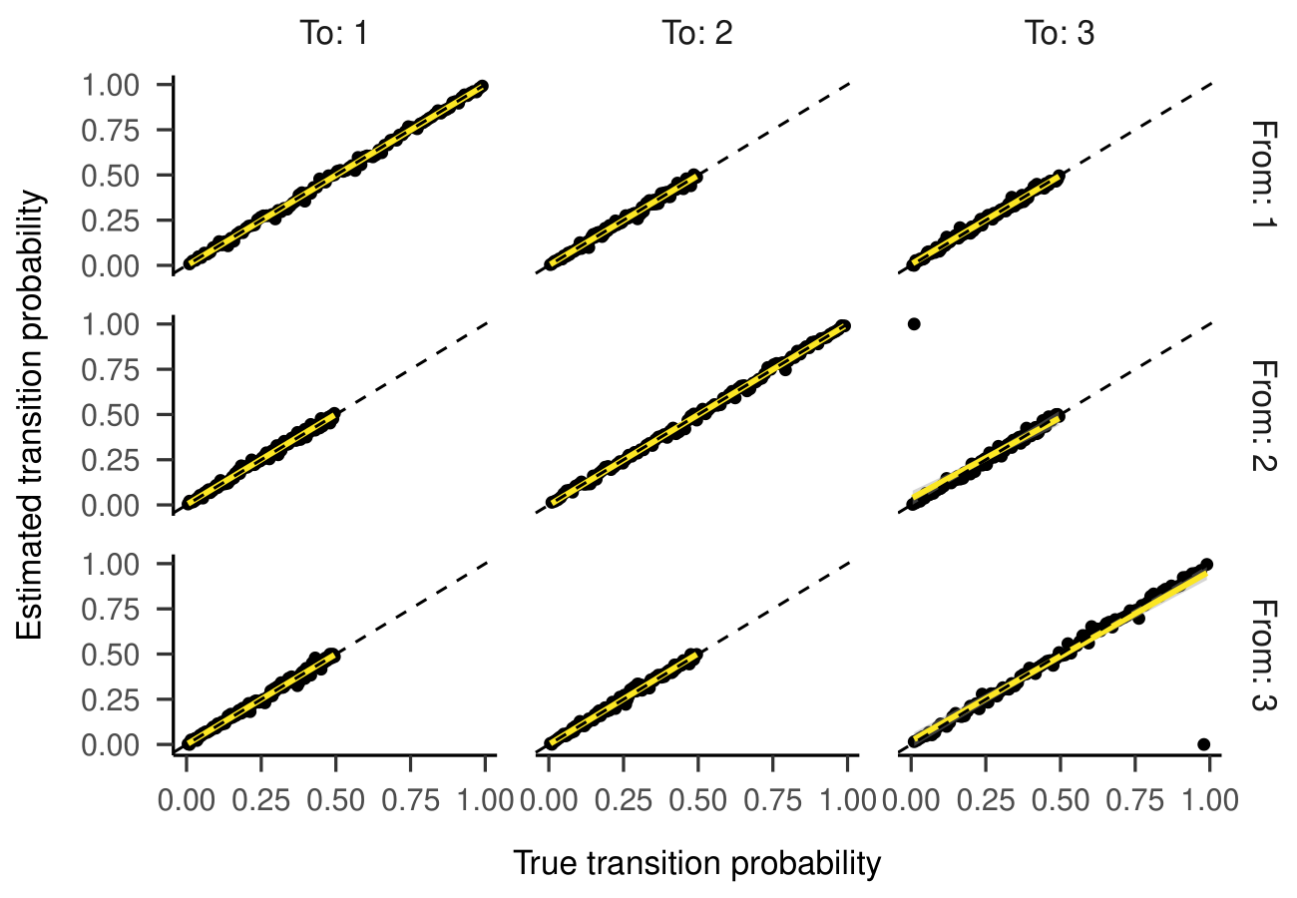
Regression Lines Between True and Estimated Transition Probabilities for the Three-State HMM in Part One of the Simulation.

**Figure 5.**
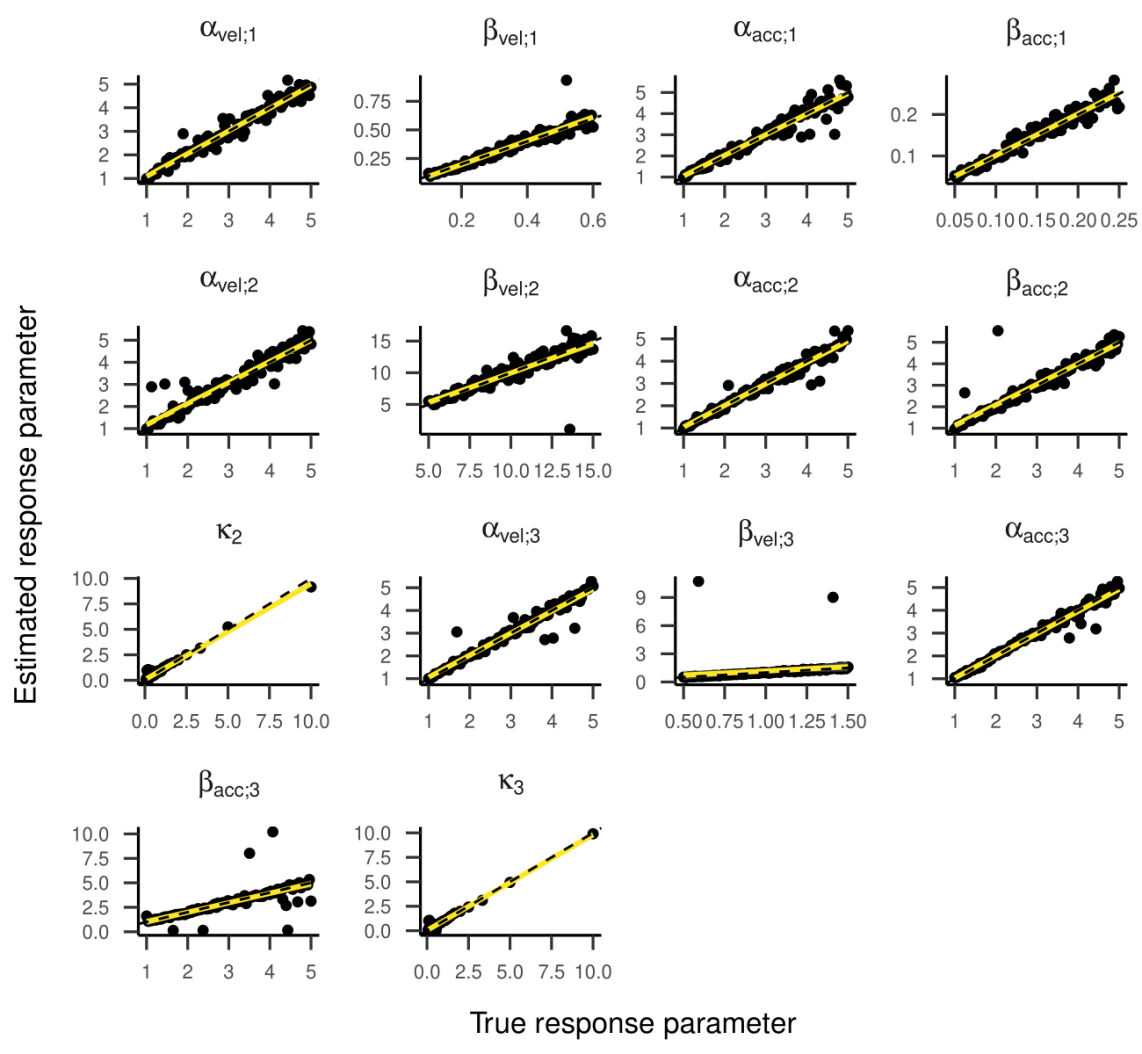
Regression Lines Between True and Estimated Response Parameters of the Three-State HMM in Part One of the Simulation.

**Figure 6.**
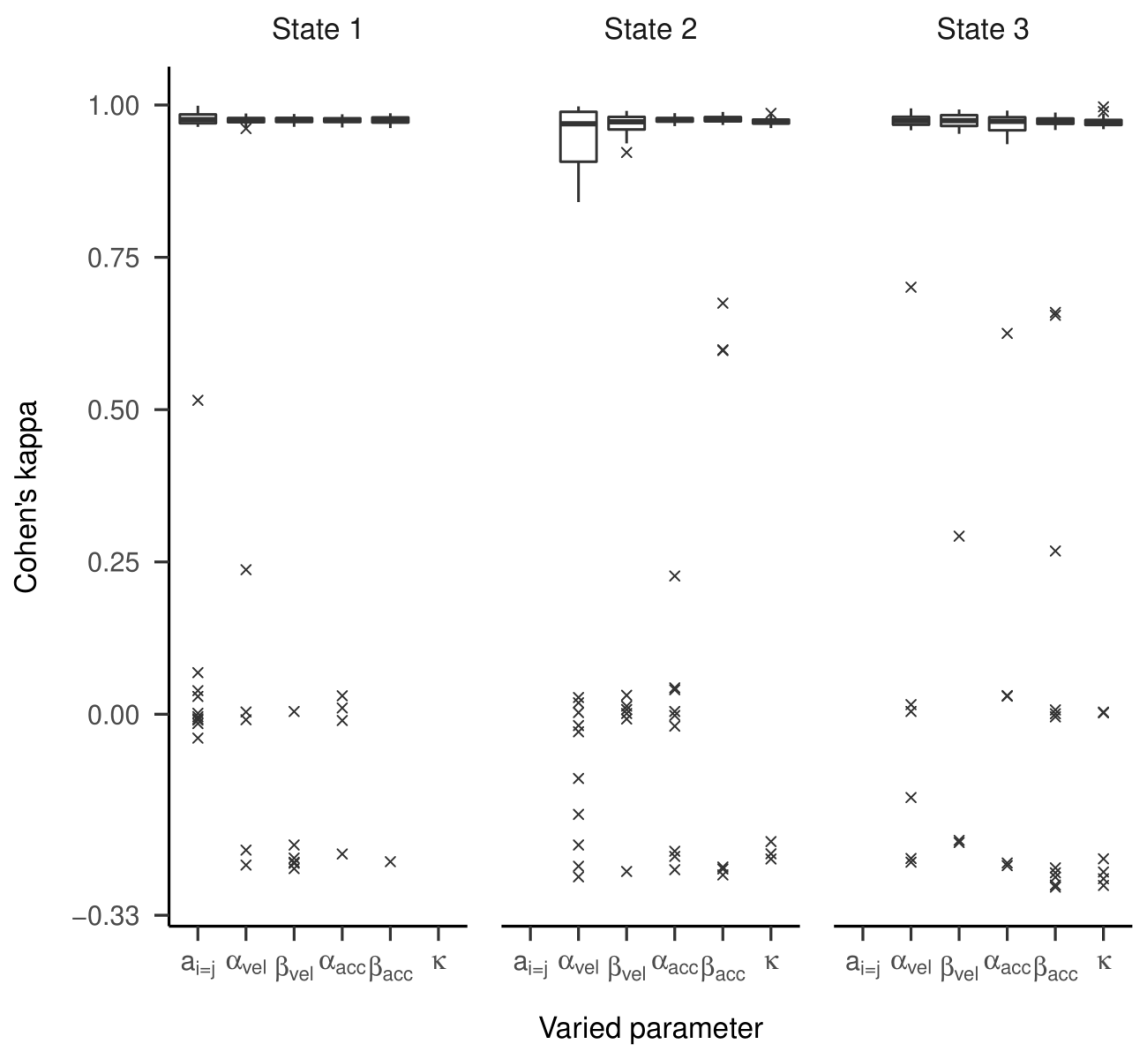
Cohen’s Kappa Depending on Which Parameter of the Three-State HMM Has Been Manipulated in Part One of the Simulation.

Inspecting the regression lines between true and estimated parameters (see Figures 4 and 5) revealed strong and unbiased linear relationships (intercepts close to zero and slopes close to one). In contrast to the two-state HMM, larger deviations and more outliers were observed. Cohen’s kappa values are presented in Figure 6. For most estimated models, the kappa values between true and estimated state sequences were above 0.95, meaning almost perfect agreement. However, for some models, we observed kappas clustered around zero or -0.33, which is far from the majority of model accuracies. An exploratory examination of these clusters suggests that state labels were switched (see supplementary material).

**Figure 7.**
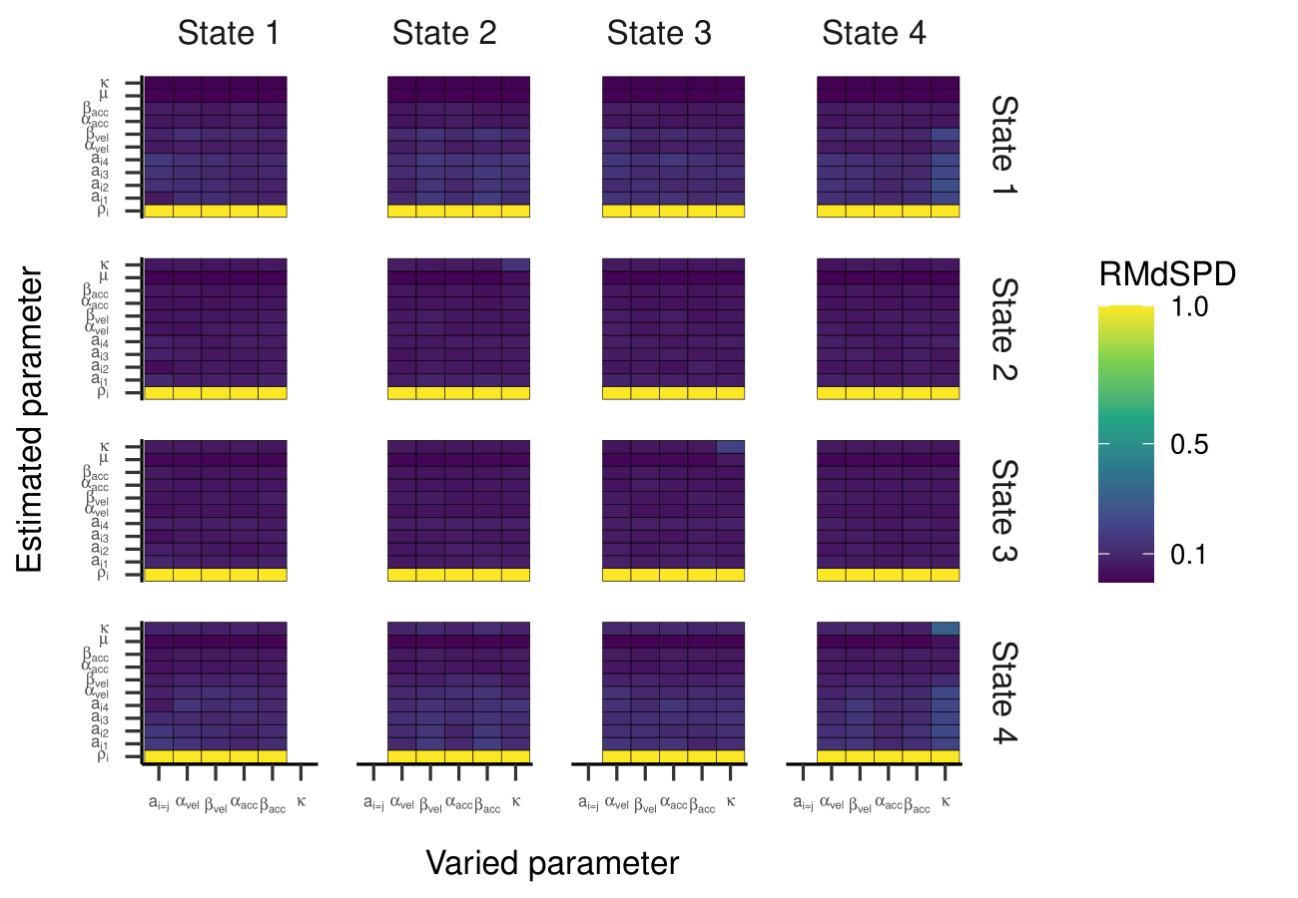
RMdSPD between true and estimated parameters of the four-state HMM in part one of the simulation.

**Figure 8.**
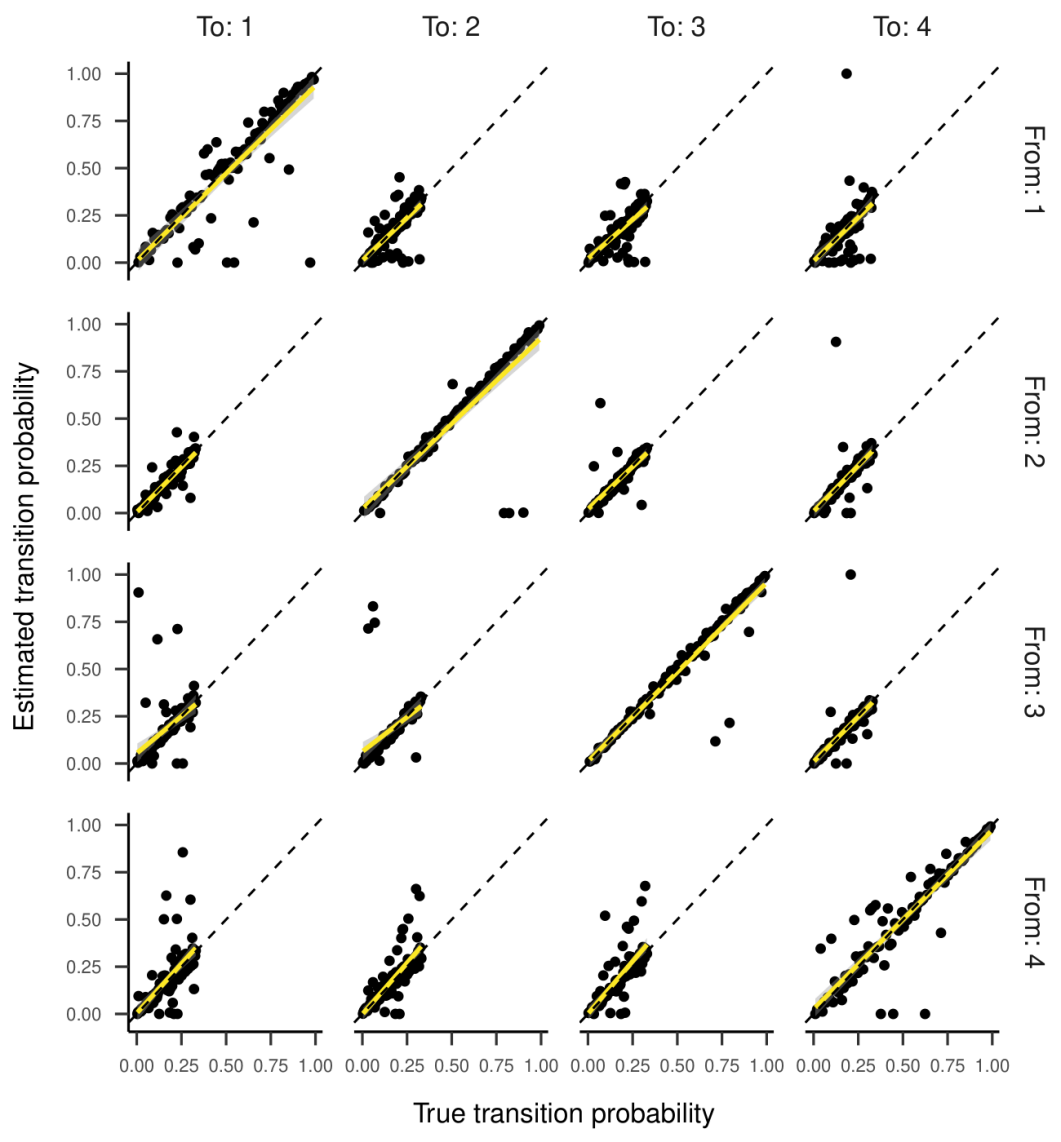
Regression Lines Between True and Estimated Response Parameters of the Four-State HMM in Part One of the Simulation (transition parameters).

**Figure 9.**
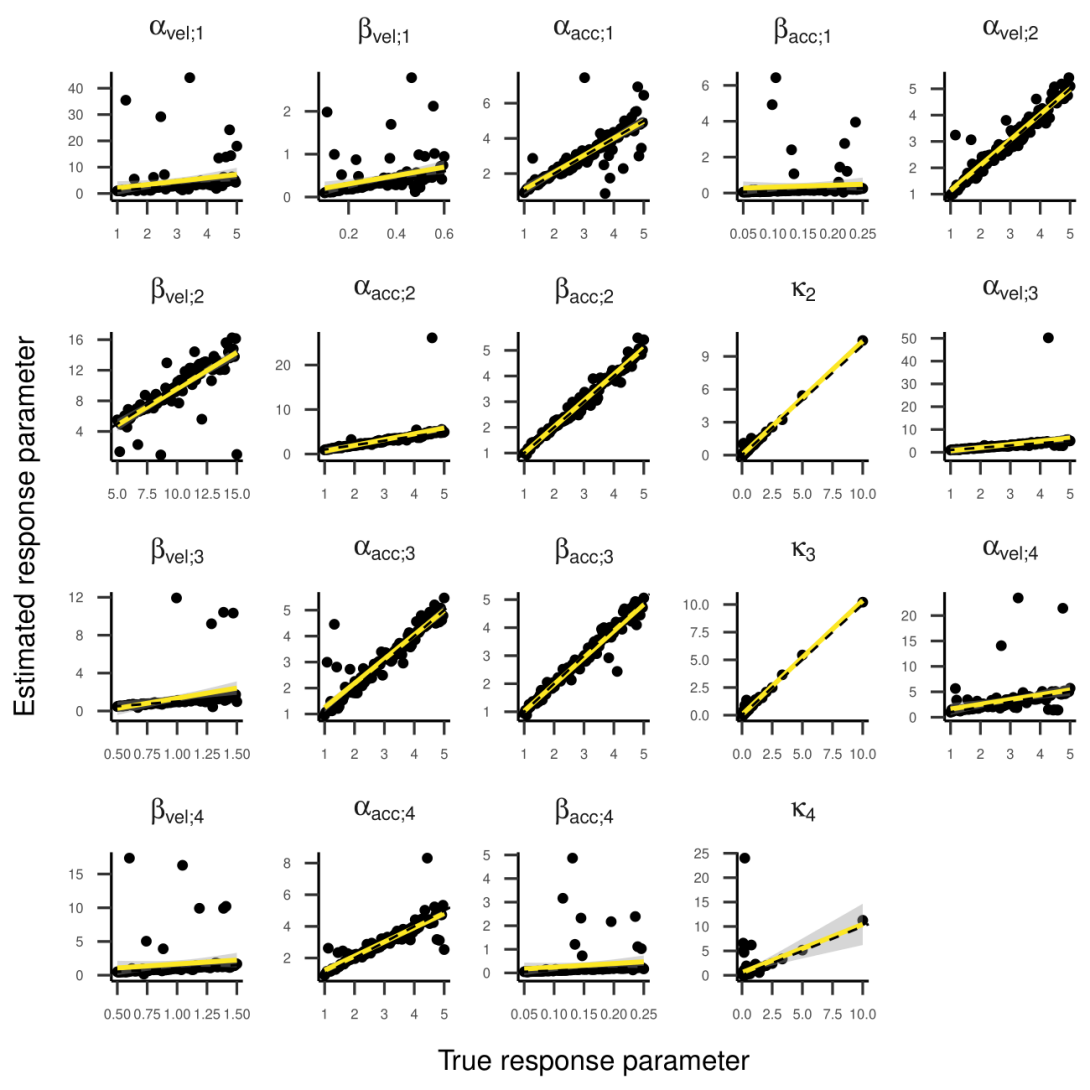
Regression Lines Between True and Estimated Response Parameters of the Four-State HMM in Part One of the Simulation.

**Figure 10.**
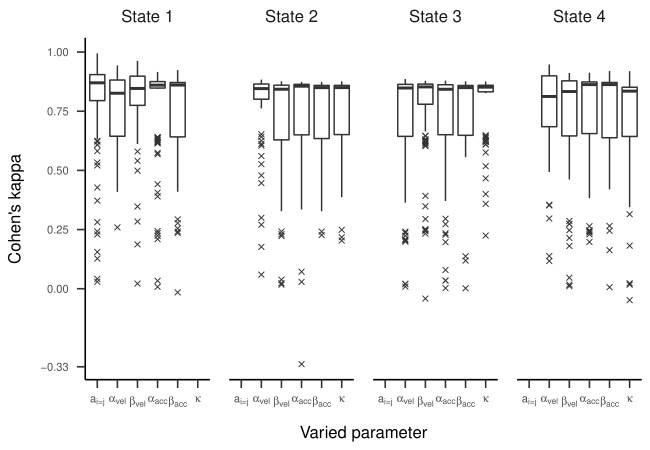
Cohen’s Kappa Depending on Which Parameter of the Four-State HMM Has Been Manipulated in Part One of the Simulation.

The RMdSPDs for the four-state HMM is shown in Figure 7. For estimated transition probabilities and α_vel_ and β_vel_ parameters in states one and four, RMdSPDs were between 0.1 and 0.5, suggesting moderate recovery. Also, estimated kappa parameters in state four were often moderately recovered when parameters in states two, three, and four were varied. Otherwise, RMdSPDs were below 0.1, indicating good recovery. Looking at Figures 8 and 9, the regression lines between true and estimated parameters exhibit strong and unbiased relationships. However, there were larger deviations and more outliers than in the previous models, especially for states one and four. Cohen’s kappa ranged mostly between 0.6 and 0.9, meaning moderate to almost perfect agreement between true and estimated state sequences (see Figure 10). Here, some outlying kappa values clustered around 0.25 and zero.

#### Sample Size and Noise Variation

**Figure 11.**
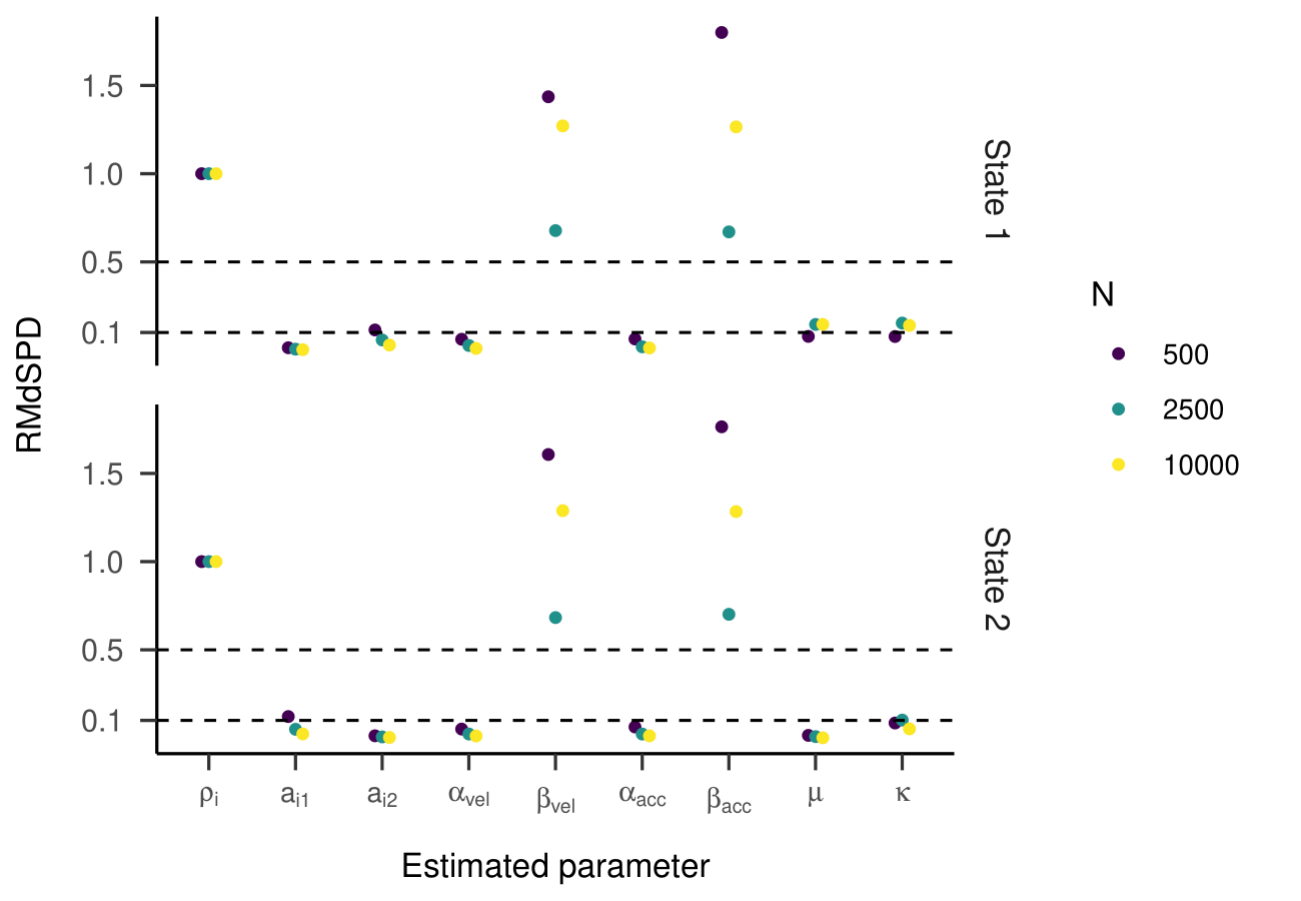
RMdSPD Between True and Estimated Parameters of the Two-State HMM in Part Two of the Simulation.

**Figure 12.**
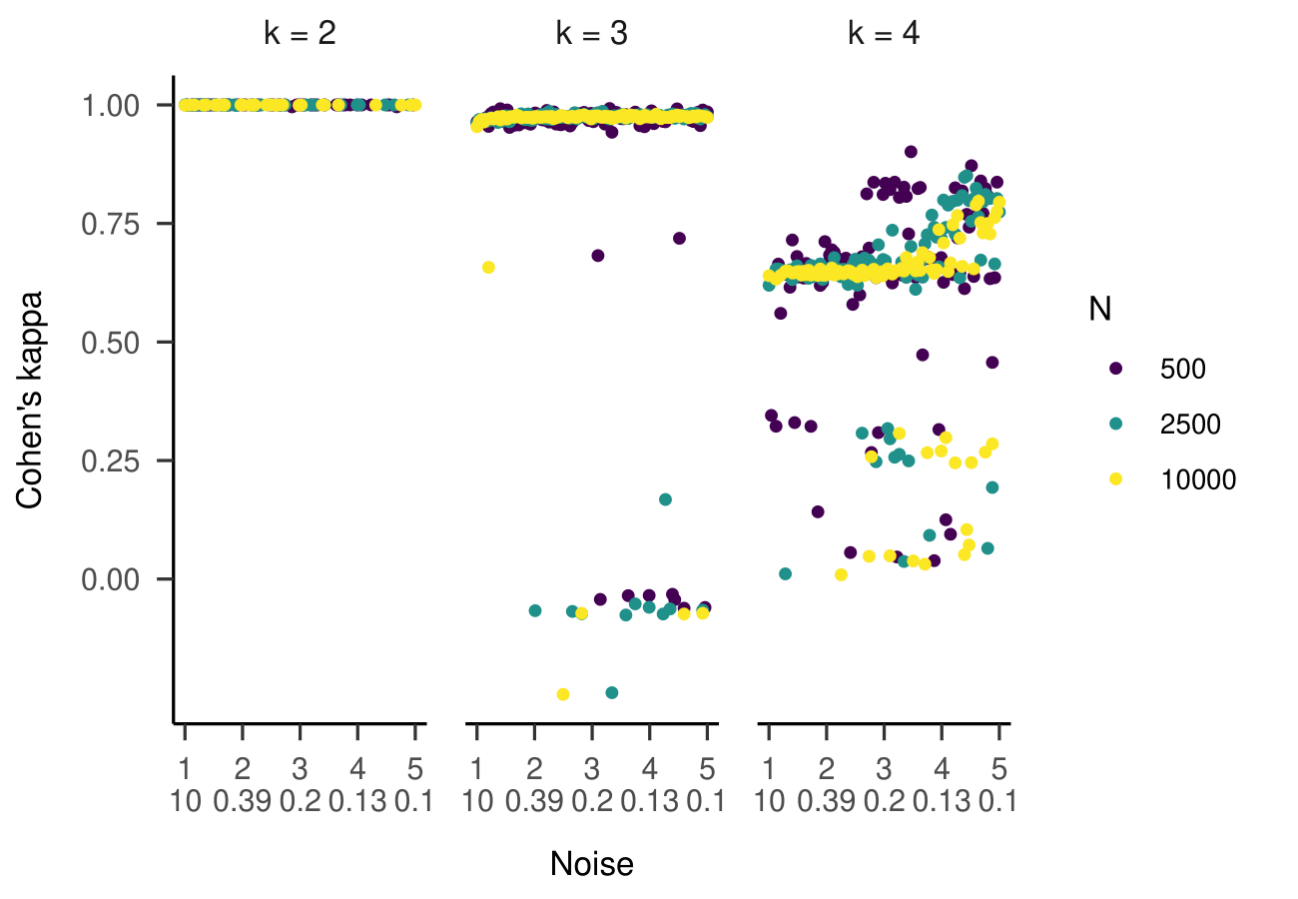
Cohen’s Kappa Depending on the Variation of Noise Added to the Simulated Data.

In the second part, we varied the sample size of the HMM and added noise to the generated data. For the two-state HMM, the RMdSPDs were above 0.5 for β_vel_ and β_acc_ in both states (see Figure 11), suggesting bad recovery. The other estimated parameters showed RMdSPDs close to or below 0.1, which means they were recovered well. Increasing the sample size seemed to improve RMdSPDs for most parameters slightly. For β_vel_ and β_acc_ in both states, models with 2500 samples had the lowest RMdSPDs. Accuracy measured by Cohen’s kappa was almost perfect with kappa values very close to one (see Figure 12, left plot).

**Figure 13.**
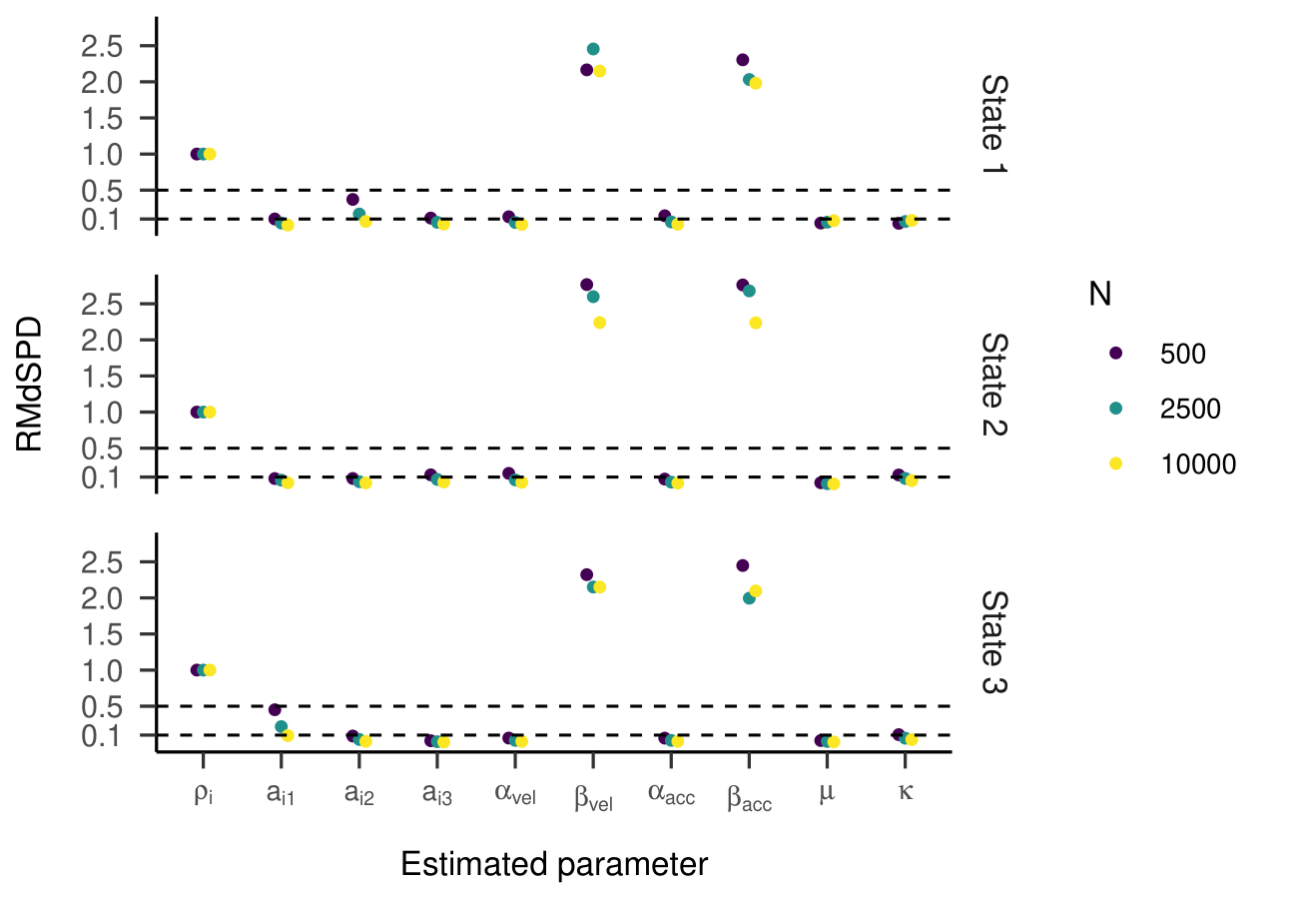
RMdSPD Between True and Estimated Parameters of the Three-State HMM in Part Two of the Simulation.

**Figure 14.**
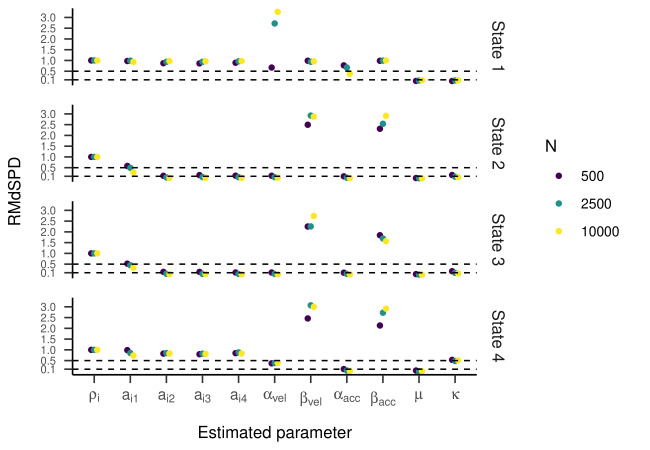
RMdSPD Between True and Estimated Parameters of the Four-State HMM in Part Two of the Simulation.

For three states, the RMdSPDs for the β_vel_ and β_acc_ were above 0.5 in all three states (see Figure 13), indicating bad recovery. Again, the other estimated parameters were below or close to 0.1, only a_12_ and a_31_ with 500 samples were closer to 0.5. For most parameters across all three states, models with higher sample sizes had lower RMdSPDs. The state recovery of the estimated models was almost perfect with most kappa values above 0.95 (see Figure 12, middle plot). Several outliers clustered around kappa values of zero and -0.33.

RMdSPDs regarding the four-state HMM are displayed in Figure 14. For states one and four, values for most parameters (including all transition probabilities) were above 0.5, suggesting bad recovery. Similarly, β_vel_ and β_acc_ in states two and three showed bad recovery. For states two and three, higher sample sizes showed slightly lower RMdSPDs. As in the previous part, most Cohen’s kappa values ranged between 0.6 and 0.9, meaning substantial to almost perfect agreement between true and estimated states (Figure 12, right plot). Multiple outliers clustered around 0.25 or zero.

### Discussion

In the simulation study, we assessed the recovery of parameters and hidden states in the generative model of gazeHMM. Simulations in part one demonstrated that the HMM recovered parameters very well when they were manipulated. Deviations from true parameters were mostly small. In the four-state model, estimated transition probabilities for state one and four deviated moderately. Moreover, the HMM estimated state sequences very accurately with decreasing accuracy for the four-state model. In the second part, noise was added to the generated data and the sample size was varied. Despite noise, the generative model was still able to recover most parameters well. However, in the four-state model, the parameter recovery for states one and four substantially decreased (even for low amounts of noise, see supplementary material). In the three- and four-state models, scale parameters of gamma distributions were poorly recovered (also even for low noise levels, see supplementary material). Increasing the sample size in the HMM slightly improved the recovery of most parameters. The state recovery of the model was slightly lowered when more states were included, but it was neither affected by the noise level nor the sample size. In the third part (included in the supplementary material), we showed that the variation in starting values used to fit the HMM did not influence parameter and state recovery. Missing data (in part four, also in the supplementary material) did not affect the parameter recovery but linearly decreased the recovery of hidden states. In all four parts, we observed clusters of outlying accuracy values. In part three, we exploratorily examined these clusters and reasoned that they can be attributed to label switching (i.e., flipping one or two state labels resolved the outlying clusters).

In general, the generative model recovers parameters and hidden states well and, thus, we conclude that it can be used in our classification algorithm. However, the recovery decreases when a fourth state (i.e., smooth pursuit) is added to the model and, especially with four states, many parameters in the HMM are vulnerable to noise. In the next sections, we will see how noise that is present in real eye movement data affects the performance of gazeHMM.

A limitation of this simulation study is that it only concerns the statistical part of the model, and investigates the ability of the model to recover the parameter values and state sequences. As such, the simulation study is an implementation as well as feasibility check of the method. It does not, however, test accuracy of the final event labels, which are determined using the modeling output and postprocessing steps. Thus, the simulation might not be entirely realistic: for example, the generative statistical model is not constrained to allow PSO events follow only saccade events, and so this feature of the process would not be accounted for in the simulation results.

## Validation Study

To validate gazeHMM, we applied the algorithm on two benchmark data sets. As starting values, we used ρ = 1/k for the initial state model as well as a_i=j_ = 0.9 and a_i̸=j_ = 0.1/k for the transition model. The values for the response model are displayed in Table 4. For a fifth eye movement event, we chose starting values that would enable the HMM to split any other event into two subevents (e.g., fixations into drift and microsaccades). In contrast to the simulation study, generating random starting values often led to bad model fits and label switching between states. To improve the fitting of the gamma distributions, velocity and acceleration signals were scaled down by factor 100, and so were the starting values for their gamma distributions.

**Table 4.**
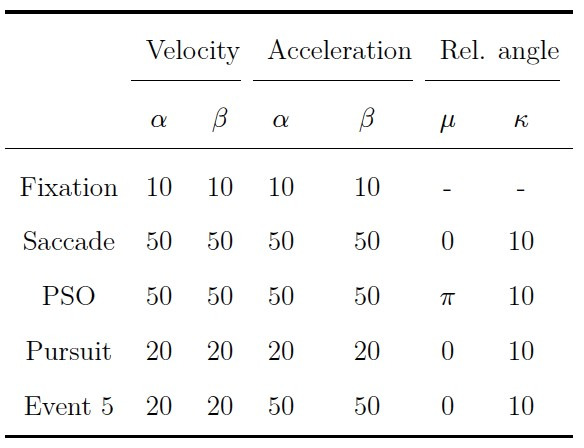
Starting Values for the Response Model in the Validation Study.

Note. Starting values for velocity and acceleration signals are shown before scaling down by factor 100. Shape parameters are denoted by α, scale parameters by β, mean parameters by μ, and concentration parameters by κ.

### Data Sets

We chose two data sets for validation: One was published in a study by Andersson et al. (1) and has been widely used for validation purposes (e.g., 38). It contains eye-tracking data from three conditions: A static condition, where subjects had to look freely at images, and two dynamic conditions, where they had to follow a constantly moving dot or objects in a video. The data were sampled with 500 Hz and two human coders (MN and RA) labeled them as belonging to six different eye movement events: Fixation, saccade, PSO, smooth pursuit, blink, or other. Andersson et al. (1) used the data to compare 10 different classification algorithms. We adopted their results to compare these 10 algorithms and the two human coders with gazeHMM. We used the original data from the study but removed two recordings from the moving dots condition because they majorly contained samples labeled as “other” or blinks. Moreover, the recordings could not be matched to the results obtained by Andersson et al. (1). Two recordings from the moving dots conditions were substantially longer than the other recordings in the condition and contained more samples than were classified by the algorithms in the study by Andersson et al. (1). Since no sample indices were available in the data set, we could not match samples from the two recordings to the labels assigned by the algorithms and therefore decided to remove them from the analysis. We do not expect the conclusions of our analyses to depend on these two data sets.

The second data set was published in Ehinger et al. (11) and has to our knowledge not yet been used for validation. Here, we only took tasks four and five out of 10 tasks because these are qualitatively different from the first data set. In task four, subjects were instructed to fixate a central target for 20 s. Task 5 was set up similarly, but subjects had to blink when they heard one out of seven beeps (with a beep duration of 100 ms and 1.5 s intervals in between). Eye movements were recorded with 500 Hz for 10 participants and 250 Hz for 5 participants due to a technical mistake (11). We used only data obtained by the EyeLink (SR Research Ltd., Ontario, Canada) eye-tracker and excluded recording using PupilLabs glasses, as wearable eye-tracker violates our methods’ definition of frame of reference (19).

### Data Analysis

Successful validation of gazeHMM was determined by two approaches: First, we applied gazeHMM with generative models containing 1-5 states to both data sets. The fits of the generative models were compared using Schwarz weights (56), a conversion of the BIC (44) into model weights. They can be interpreted as the probability of a model having generated the data compared to the competing models. For the static condition in the Andersson et al. (1) data set, we expected the generative model with three states (fixation, saccade, and PSO), and for the dynamic conditions the model with four states (incl. smooth pursuit) to display the highest Schwarz weight. Regarding the Ehinger et al. (11) data set, we assumed that the one-state model (only fixation) would show the highest weights for both tasks.

The algorithm was applied separately to every subject, condition/task. For the Andersson et al. (1) data set, all generative models were successfully fitted, whereas, for the Ehinger et al. (11) data set, it was only 780 out of 900 models (87%, 60 models per task). The erroneous model fits in the Ehinger et al. (11) data occurred when applying HMMs with three states or more. We attribute them to low variance in the data (i.e., it is difficult to fit data where subjects only fixate the same location with an HMM that assumes three or more states/events).

Second, we compared gazeHMM to other algorithms and human coders. We applied our algorithm with a three-state generative model to the static condition in the Andersson et al. (1) data set, and with a four-state model to the dynamic conditions. For comparison criteria, we followed Andersson et al. (1): We calculated the RMSD of event durations and counts between all algorithms and the average of the two human coders.

Our results differ slightly from the original study because we excluded two recordings (leading to fewer events) and calculated the event durations as Dur(e) = max(**t**_e_) − max(**t**_e−1_), where **t_e _**is the vector of sample time stamps for the event e. Cohen’s kappa was calculated for each event as the binary agreement between the algorithms and the average of the human coders. Lastly, the overall disagreement indicated which samples were classified differently by the algorithms compared to the average of the human coders across all events. The human coders were compared directly to each other.

### Results

#### Model Comparison

**Figure 15.**
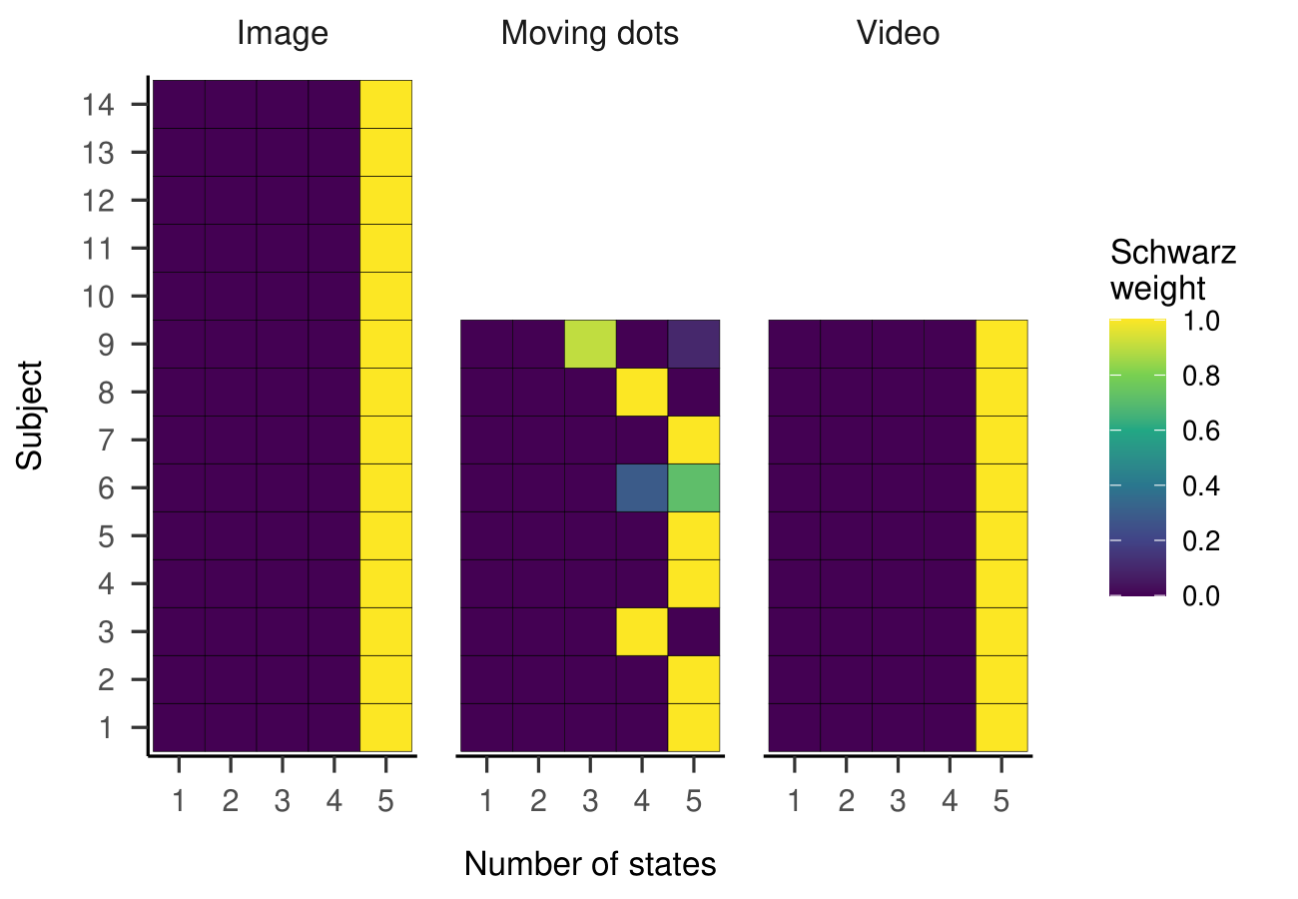
Schwarz Weights Displayed for Each Subject and HMMs With Different Numbers of States.

Examining the Schwarz weights displayed in Figure 15, we observed that the five-state generative model showed the highest weights in all three conditions. Only in the moving dots condition, two subjects displayed the highest weights for the four-, and one subject for the three-state model. In sum, we concluded that the five-state generative model has most likely generated the Andersson et al. (1) data, opposing our expectations. Because the Ehinger et al. (11) data set showed a similar pattern, we included the results for this data in the supplementary material.

A recent model recovery study showed that the BIC tended to prefer overly complex HMMs when they were misspecified (e.g., the conditional independence assumption was violated; 39). Instead, the integrated completed likelihood (ICL) criterion

Biernacki et al., 5 performed better at choosing the correct data-generating model. Therefore, we post hoc computed the weighted ICL criterion (analogous to Schwarz weights) for the models fitted to the Andersson et al. (1) data set. Using the ICL as the model selection criterion yielded very similar results to the BIC (see supplementary material). The preference for the five-state generative model was even more consistent across conditions and subjects.

#### Comparison to Other Algorithms

As displayed in Table 5, gazeHMM showed a relatively low RMSD for fixations in the static condition compared to the other algorithms that were applied to the Andersson et al. (1) data set. The lower RMSD for fixations indicated more similar classification to the human coders in terms of their mean and SD duration as well as the number of classified fixations. Oppositely, for fixations in the dynamic conditions, the RMSD of gazeHMM was one of the highest among the compared algorithms, suggesting substantial differences to the human coders. It can be seen that gazeHMM classified a much larger number of fixations with very short durations. For saccades, gazeHMM had a relatively high RMSD for the static condition but the lowest RMSD for the moving dots condition, and a moderate value for the video condition (see Table 6). The deviation was mostly because gazeHMM classified a higher number of saccades than the human coders. Only two other algorithms classified PSOs (NH and LNS; 36; 28). Here, gazeHMM showed a consistently higher RMSD than LNS and lower RMSD than NH (see Table 7). Our algorithm classified shorter and more PSOs than the human coders. No other algorithm parsed smooth pursuits, but the RMSD for gazeHMM was higher than among human coders (see Table 8). Again, it classified a much larger number of smooth pursuits with short durations.

Table 9 contains the sample-to-sample agreement between the algorithms and human coders measured by Cohen’s kappa. For fixations, gazeHMM showed one of the highest agreements for static and the highest agreements for dynamic data. The absolute agreement was substantial for the static and slight to fair for the dynamic conditions (26). For saccades, gazeHMM had the lowest agreement for the static condition and moderate agreement for the dynamic conditions. In absolute terms, the agreement was fair to moderate. Concerning PSOs, gazeHMM showed higher agreement than NH in the image and video conditions but consistently lower agreement compared to LNS. The absolute agreement was slight (image) to moderate (video). Lastly, the agreement for smooth pursuit was lower compared to the human coders and fair in absolute values.

**Figure 16.**
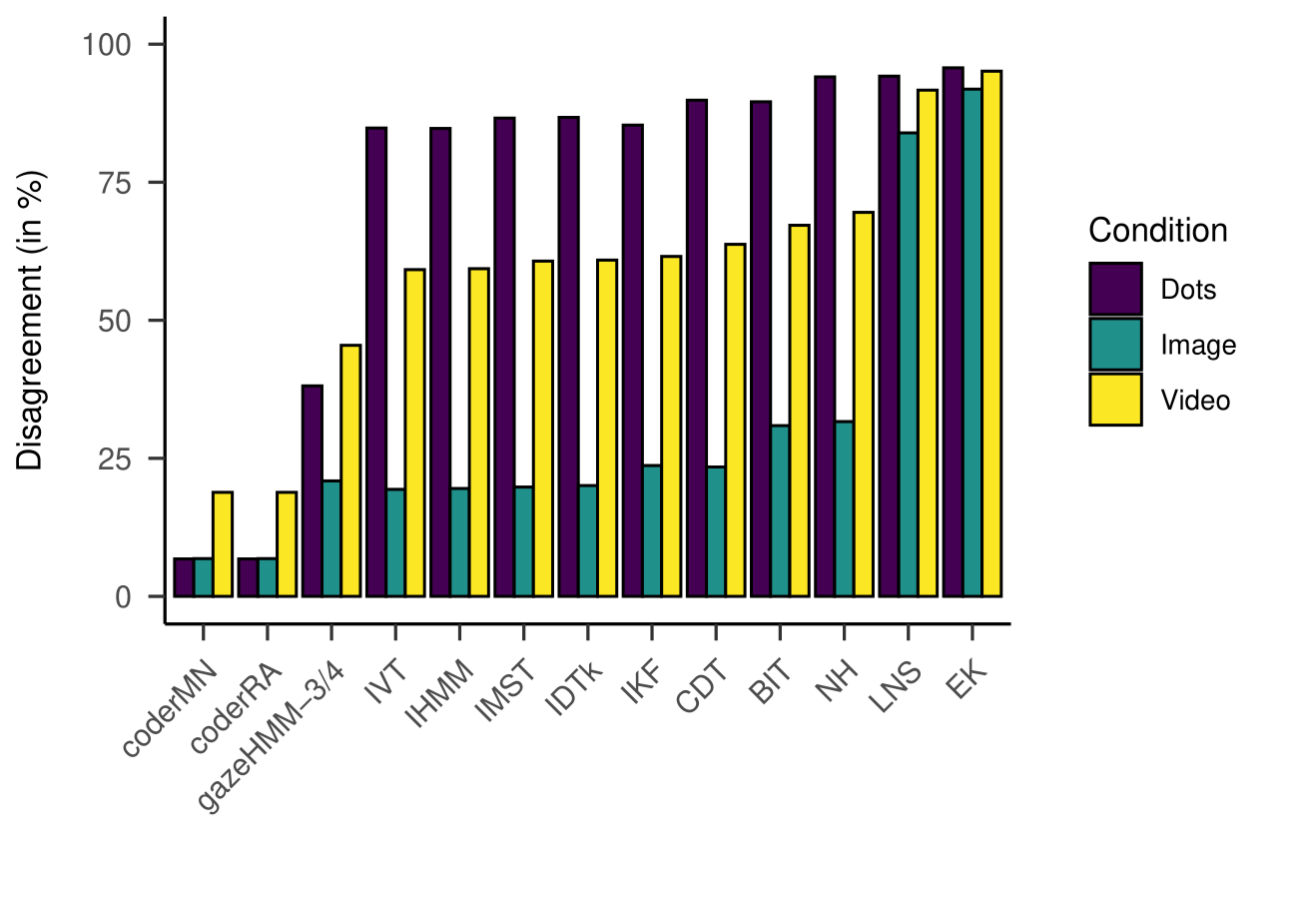
Disagreement Between Algorithms and Human Coders for Different Conditions

Note. gazeHMM-3/4 classified three events for image data and four events for moving dots/video data. Algorithms are displayed in order according to mean disagreement over conditions (least/left to highest/right).

Concerning overall disagreement, Figure 16 shows that gazeHMM had less disagreement to the human coders across all events for the dynamic conditions. For the static condition, we interpreted the difference to most other algorithms as slight (Med(∆) = 2.65%), but for the dynamic conditions, as substantial (video: Med(∆) = 17.19%) and large (dots: Med(∆) = 50.04%).

**Figure 17.**
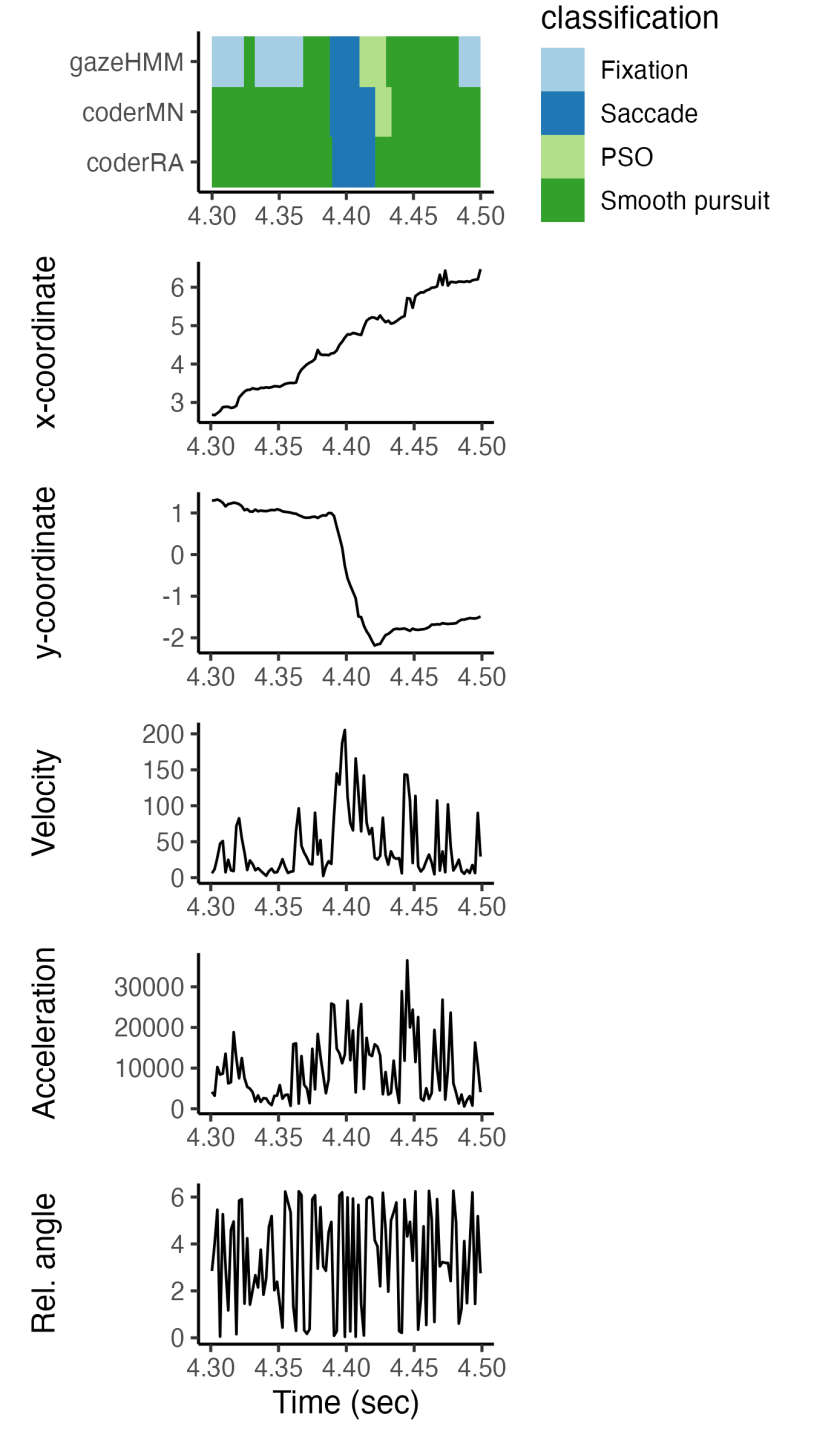
Classification of Example Data by Andersson et al. (1).

Note. Data displayed as x-, and y-coordinates (in deg, upper two panels), velocity (in deg/s, middle panel), acceleration (in deg/s2, fourth panel), and sample-to-sample (relative) angle (in radians, bottom panel). The top-most panel displays event classification by gazeHMM,coderMN, and coderRA, highlighted by color.

To explore which events gazeHMM classified differently than the average human coder, we looked at the confusion matrix between the two (see Table 10). It can be seen that gazeHMM classified many fixation samples as smooth pursuit samples and vice versa. Moreover, it confused many PSOs with saccade samples. The heuristic to detect blinks seemed to work successfully since gazeHMM classified most blink samples in agreement with human coding and only a minor part was mistaken for saccades. Inspecting an example of gaze data classified by gazeHMM compared to human coding leads to a similar notion: Figure 17 illustrates that gazeHMM is rapidly switching between classifying fixations and smooth pursuits, whereas the human coder identified one large smooth pursuit event. In the example, gazeHMM also disagrees with the human coder regarding the start of the PSO.

**Table 5.**
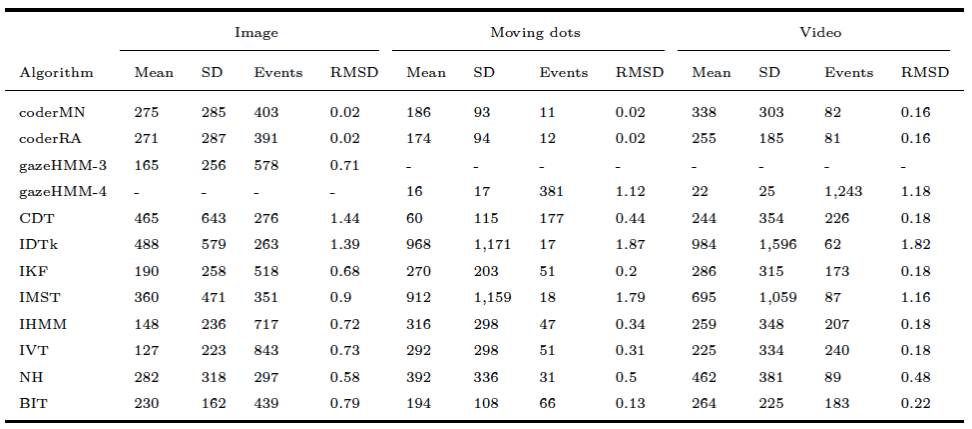
Fixation Duration Descriptives and RMSD Between Algorithms and Human Coders.

Note. Durations are displayed in milliseconds. gazeHMM-3 classified three and gazeHMM-4 classified four events. RMSD = root mean square deviation. Table design adapted from Andersson et al. (1).

**Table 6.**
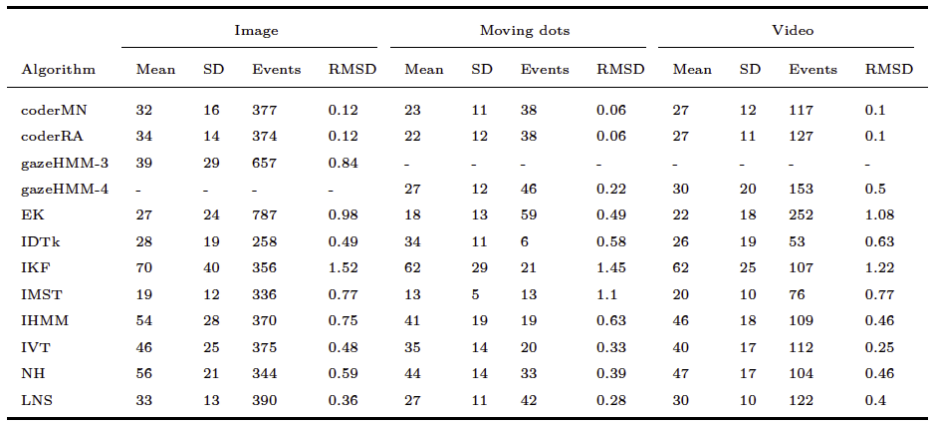
Saccade Duration Descriptives and RMSD Between Algorithms and Human Coders.

Note. Durations are displayed in milliseconds. gazeHMM3 classified three and gazeHMM-4 classified four events. RMSD = root mean square deviation. Table design adapted from Andersson et al. (1).

**Table 7.**
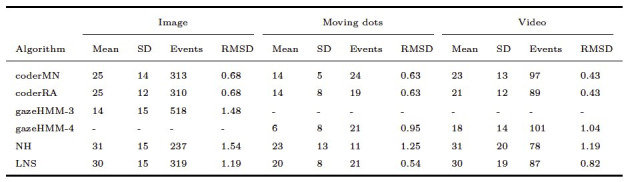
PSO Duration Descriptives and RMSD Between Algorithms and Human Coders

Note. Durations are displayed in milliseconds. gazeHMM-3 classified three and gazeHMM-4 classified four events. RMSD = root mean square deviation. Table design adapted from Andersson et al. (1).

**Table 8.**
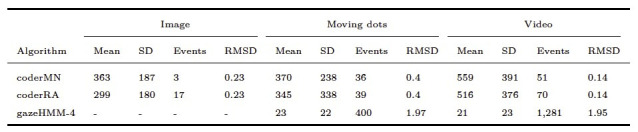
Smooth Pursuit Duration Descriptives and RMSD Between gazeHMM and Human Coders.

Note. Durations are displayed in milliseconds. gazeHMM-3 classified three and gazeHMM-4 classified four events. RMSD = root mean square deviation. Table design adapted from Andersson et al. (1).

**Table 9.**
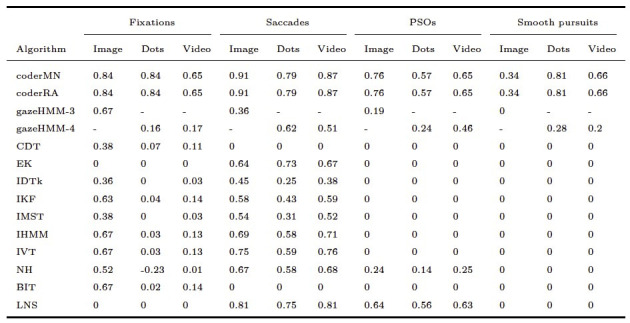
Cohen's Kappa Between Human Coders and Algorithms for Different Conditions and Events.

Note. gazeHMM-3 classified three and gazeHMM-4 classified four events. Table design adapted from Andersson et al. (1).

**Table 10.**
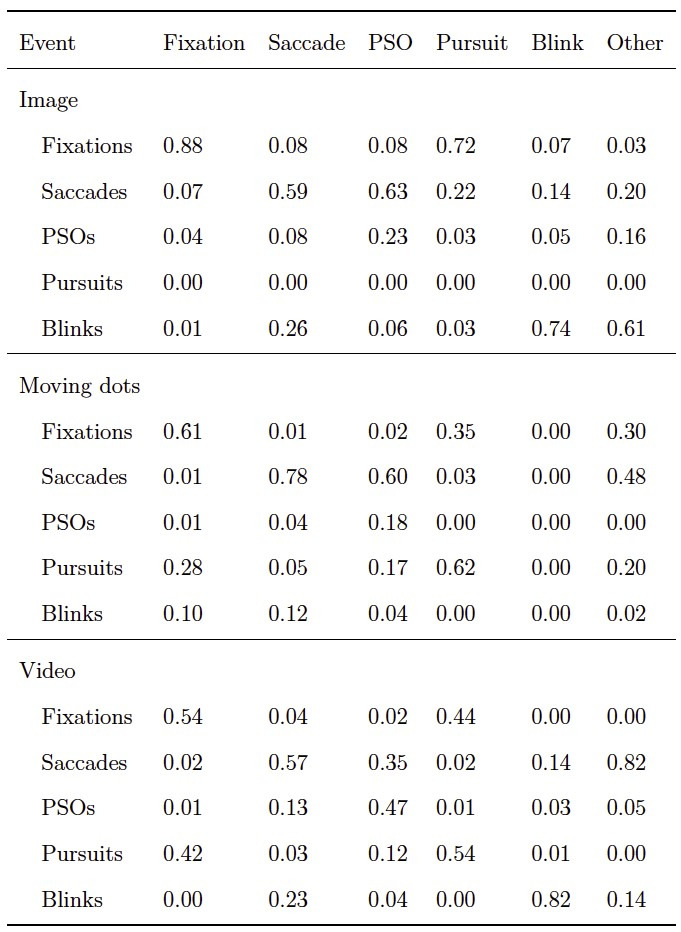
Confusion Matrix Between gazeHMM (rows) and Human Coders (columns) for Different Conditions.

Note. gazeHMM classified four events and blinks. Values indicate proportions of samples where gazeHMM and human coders agree divided by the total number of samples classified by the human coders for each event (i.e., columns sum to one).

## General Discussion

In this report, we presented gazeHMM, a novel algorithm for classifying gaze data into eye movement events. The algorithm models velocity, acceleration, and sample-to-sample angle signals with gamma distributions and a mixture of von Mises and a uniform distribution. An HMM serves as the generative model of the algorithm and classifies the gaze samples into fixations, saccades, and optionally PSOs, and/or smooth pursuits. We showed in a simulation study that the generative model of gazeHMM recovered parameters and hidden state sequences well. However, adding a fourth event (i.e., smooth pursuit) to the model and introducing even small amounts of noise to the generated data led to decreased parameter recovery. Importantly, however, it did not lead to decreased hidden state recovery. Thus, the classification of the generative model should not be negatively affected by noise. Furthermore, we applied gazeHMM with different numbers of states to benchmark data by Andersson et al. (1) and compared the model fit. The model comparison revealed that a five-state HMM had consistently most likely generated the data. This result opposed our expectation that a three-state model would be preferred for static and a four-state model for dynamic data. When comparing gazeHMM against other algorithms, gazeHMM showed mostly good agreement to human coding. On one hand, it outperformed the other algorithms in the overall disagreement with human coding for dynamic data. On the other hand, gazeHMM confused a lot of fixations with smooth pursuits, which led to rapid switching between the two events. It also tended to mistake PSO samples as belonging to saccades.

Considering the results of the simulation study, it seems reasonable that adding the smooth pursuit state to the HMM decreased parameter and state recovery: It is the event that is overlapping most closely with another event (fixations) in terms of velocity, acceleration, and sample-to-sample angle. The overlap can cause the HMM to confuse parameters and hidden states. The decrease in parameter recovery (especially for scale parameters) due to noise shows that the overlap is enhanced by more dispersion in the data. The scale parameters might be particularly vulnerable to extreme data points. Despite these drawbacks, the recovery of the generative model in gazeHMM seems very promising. The simulation study gives also an approximate reference for the maximum recovery of hidden states that can be achieved by the HMM (Cohen’s kappa values of ~1 for two, ~0.95 for three, and ~0.8 for four events).

The model comparison on the benchmark data suggested that the generative model in gazeHMM is not yet optimally specified for eye movement data. There are several explanations for this result:

The model subdivided some events into multiple events, or found additional patterns in the data that do not fit the other four events the model was built for. Eye movement events can be divided into subevents. For example, fixations consist of drift and tremor movements (10) and PSOs encompass dynamic, static, and glissadic over- and undershoots (28). A study on a recently developed HMM algorithm supports this explanation: Houpt et al. (22) applied the unsupervised BP-AR-HMM algorithm to the Andersson et al. (1) data set and classified more distinct states than the human coders. Some of the states classified by BP-AR-HMM matched the same event coded by humans. Since the subevents are usually not interesting for users of classification algorithms, the ability of HMMs to classify might limit their ability to generate eye movements.

Model selection criteria are generally not appropriate for comparing HMMs with different numbers of states. This argument has been discussed in the field of ecology (see 31), where studies found that selection criteria preferred models with more states than expected (similar to the result of this study; e.g., 27). Li and Bolker (31) explain this bias with the simplicity of the submodels in HMMs: Initial state, transition, and response models for each state are usually relatively simple. When they do not describe the processes in the respective states accurately, the selection criteria compensate for that by preferring a model with more states. Thus, there are not more latent states present in the data, but the submodels of the HMM are misspecified or too simple, potentially leading to spurious, extra, states being identified in the model selection process, see discussion and potential solutions in Kuijpers et al. (25). Correcting for model misspecifications led to a better model recovery in studies on animal movements (27; 31). However, Pohle et al. (39) showed in simulations that the ICL identified the correct model despite several misspecifications. It has to be noted that the study by Pohle et al. (39) only used data generating models with two states, so it needs to be verified whether this approach will work in the larger models that are being studied here.

The submodels of gazeHMM were misspecified. Pohle et al. (39) identified two scenarios in which model recovery using the ICL did not give optimal results: Outliers in the data and inadequate distributions in the response models. Both situations could apply to gazeHMM and eye movement data: Outliers occur frequently in eye-tracking data due to measurement error. Choosing adequate response distributions in HMMs is usually difficult and can depend on the individual and task from which the data are obtained (27). Moreover, gazeHMM only estimated intercepts for all parameters and thus, no time-varying covariates were included (cf. 31). This aspect could indeed oversimplify the complex nature of eye movement data.

Comparing gazeHMM to other algorithms on benchmark data showed that gazeHMM showed good agreement with human coders. However, the evaluation criteria (RMSD of event durations, sample-to-sample agreement, and overall disagreement) yielded different results. The fact that gazeHMM outperformed all other algorithms regarding the overall disagreement can be because it is the only algorithm classifying all five events the human coders classified; algorithms that do not classify certain type of even will, by definition, disagree with human coders on samples that they classified as such. As the number of samples in different events depending on the stimuli (e.g., a lot of smooth pursuit in moving dots condition but virtually none in static images), different methods might be penalized differently depending on the condition and type of event they do not classify. Nevertheless, Cohen’s kappa values of 0.67 (fixations - image) or 0.62 (saccades - moving dots) indicate substantial agreement to human coders, especially in light of the maximum references from the simulation study. At this point, it is important to mention that human coding should not be considered a gold standard in event classification: Hooge et al. (21) observed substantial differences between coders and within coders over time. Despite these differences, they recommend comparisons to human coding to demonstrate the performance of new algorithms and to find errors in their design.

### Advantages of gazeHMM

Given the four proposed goals that gazeHMM should fulfill, we can draw the following conclusions: Even though gazeHMM does require some parameter settings (in the pre- and postprocessing), it estimates many parameters adaptively from the data; as a result, compared to many other algorithms, it reduces the influence of human judgement and researcher decisions on the classification result. The parameters are merely included to compensate for the drawbacks of the generative model and their default values should be appropriate for most applications. A major advantage of gazeHMM is that it does not require human-labeled data as input. Instead, it estimates all parameters and hidden states from the data. Since human coding is quite laborious, difficult to reproduce, and by times inconsistent (as noted earlier, 21), this property makes gazeHMM a good alternative to other recently developed algorithms that require human coded input (4; 38; 59). This could also explain why the agreement to human coding is lower for gazeHMM than for algorithms that learn from human-labeled data. Another advantage of gazeHMM is its ability to classify four eye movement events, namely fixations, saccades, PSOs, and smooth pursuit. Whereas most algorithms only parse fixations and saccades (1), few classify PSOs (e.g., 59), and even less categorize smooth pursuits (e.g., 38). However, including smooth pursuits in gazeHMM led to some undesirable classifications on benchmark data, resulting in rapid switching between fixation and smooth pursuit events. Therefore, we recommend using gazeHMM with four events only for exploratory purposes. Without smooth pursuits, we consider gazeHMM’s classification as appropriate for application. Lastly, its implementation in R using depmixS4 (53) should make gazeHMM a tool that is easy to use and customize for individual needs.

To conclude, our methods shows promising results in terms of ability to classify various eye movement events, does not require previously labeled data, and reduces the number of arbitrary settings determined by the researcher. As such, in case the ultimate goal is event classification, the method is a good candidate for initial rough estimate of the event classification, which can be further inspected and refined, if necessary. Compared to other approaches, the method is also easily extensible and modifiable, allows for model comparison, and as such offers applications where broadening our understanding of eye movement is of primary interest instead of the event classification itself.

### Future Directions

Despite its advantages, there are several aspects in which gazeHMM can be improved: First, a multivariate distribution could be used to account for the correlation between velocity and acceleration signals (for examples, see 2). Potential problems of this approach might be choosing the right distribution and convergence issues (due to a large number of parameters). Another option to model the correlation could be to include one of the response variables as a covariate of the other.

Second, instead of the gamma being the generic (and potentially inappropriate) response distribution, a non-parametric approach could be used: Langrock et al. (27) use a linear combination of standardized B-splines to approximate response densities, which led to HMMs with fewer states being preferred. This approach could potentially combat the problem of unexpectedly high-state HMMs being preferred for eye movement data but would also undermine the advantages of using a parametric model.

Third, one solution to diverging results when comparing gazeHMM with different events could be model averaging: Instead of using the maximum posterior state probability of each sample from the preferred model, the probabilities could be weighted according to a model selection criterion (e.g., Schwarz weight) and averaged. Then, the maximum averaged probability could be used to classify the samples into events. This approach could lead to a more robust classification because it reduces the overconfidence of each competing model and easily adapts to new data (analogous to Bayesian model averaging; 20). However, the model comparison for gazeHMM often showed extreme weights for a five-state model, which would lead to a very limited influence of the other models in the averaged probabilities.

Fourth, including covariates of the transition probabilities and response parameters could improve the fit of gazeHMM on eye movement data. As pointed out earlier, just estimating intercepts of parameters could be too simple to model the complexity of eye movements. A candidate for such a covariate might be a periodic function of time (31) which could, for instance, capture the specific characteristics of saccades, e.g., the tendency of increasing velocity at the start of the saccade and decreasing velocity at the end of the saccade. Whether covariates are improving the fit of submodels to eye movement data could in turn be assessed by inspecting pseudo-residuals and autocorrelation functions (60).

Fifth, to avoid rapid switching between fixations and smooth pursuits as well as unreasonably short saccades, gazeHMM could explicitly model the duration of events. This can be achieved by setting the diagonal transition probabilities to zero and assign a distribution of state durations to each state (7). Consequently, the duration distributions of fixations and smooth pursuits could differ from saccades and PSOs. This extension of the HMM is also called the hidden semi-Markov model and has been successfully used by Mihali et al. (35) to classify microsaccades. Drawbacks of this extension are higher computational costs and difficulties with including covariates (60).

Lastly, allowing constrained parameters in the HMM could replace some of the postprocessing steps in gazeHMM. This could potentially be achieved by using different response distributions or parameter optimization methods. Moreover, switching from the maximum likelihood to the Markov chain Monte Carlo (Bayesian) framework could help to avoid convergence problems with constrained parameters, but would also open new research questions about suitable priors for HMM parameters in the eye movement domain, efficient sampling plans, accounting for label switching, and computational efficiency, naming only a few.

### Conclusion

In the previous sections, we developed and tested a generative, HMM-based algorithm called gazeHMM. Both a simulation and validation study showed that gazeHMM is a suitable algorithm for simulating, understanding and classifying eye movement events. For smooth pursuits, the classification is not optimal and thus not yet recommended. On one side, the algorithm has some advantages over concurrent event classification algorithms, not relying on human-labeled training data being the most important one. On the other side, it is not able to identify expected events in model comparisons. The current model constitutes a proof-of-principle that a generative, maximum-likelihood based approach can provide interpretable and reliable results that are at least as good as other approaches under some circumstances. The largest advantage of this approach is however that it provides the possibility to rigorously test progress in developing extensions and improvements.

### Ethics and Conflict of Interest

The author(s) declare(s) that the contents of the article are in agreement with the ethics described in http://biblio.unibe.ch/portale/elibrary/BOP/jemr/ethics.html and that there is no conflict of interest regarding the publication of this paper.

### Acknowledgements

Šimon Kucharský was supported by the NWO (Nederlandse Organisatie voor Wetenschappelijk Onderzoek) grant no. 406.10.559.

We would like to thank Daan van Renswoude, Maartje Raijmakers, and Maximilian Maier for their helpful comments on earlier versions of this paper. Furthermore, we would like to acknowledge feedback by Karel Veldkamp and Phil Norberts in the early stage of this project.
